# Environmental trade-offs of direct air capture technologies in climate change mitigation toward 2100

**DOI:** 10.1038/s41467-022-31146-1

**Published:** 2022-06-25

**Authors:** Yang Qiu, Patrick Lamers, Vassilis Daioglou, Noah McQueen, Harmen-Sytze de Boer, Mathijs Harmsen, Jennifer Wilcox, André Bardow, Sangwon Suh

**Affiliations:** 1 National Renewable Energy Laboratory, 15013 Denver W Pkwy, Golden, CO 80401 USA; 2 Bren School of Environmental Science and Management, 2400 University of California, Santa Barbara, CA 93117 USA; 3 Copernicus Institute of Sustainable Development, Utrecht University, Princetonlaan 8a, 3584 CS Utrecht, the Netherlands; 4 PBL Netherlands Environmental Assessment Agency, PO Box 30314, 2500 GH The Hague, the Netherlands; 5 Chemical and Biomolecular Engineering Department, University of Pennsylvania, Philadelphia, PA 19104 USA; 6 Institute of Energy and Climate Research - Energy Systems Engineering (IEK-10), Forschungszentrum Jülich GmbH, Jülich, Germany; 7 Energy and Process Systems Engineering, ETH Zurich, 8092 Zurich, Switzerland

**Keywords:** Climate-change mitigation, Environmental impact

## Abstract

Direct air capture (DAC) is critical for achieving stringent climate targets, yet the environmental implications of its large-scale deployment have not been evaluated in this context. Performing a prospective life cycle assessment for two promising technologies in a series of climate change mitigation scenarios, we find that electricity sector decarbonization and DAC technology improvements are both indispensable to avoid environmental problem-shifting. Decarbonizing the electricity sector improves the sequestration efficiency, but also increases the terrestrial ecotoxicity and metal depletion levels per tonne of CO_2_ sequestered via DAC. These increases can be reduced by improvements in DAC material and energy use efficiencies. DAC exhibits regional environmental impact variations, highlighting the importance of smart siting related to energy system planning and integration. DAC deployment aids the achievement of long-term climate targets, its environmental and climate performance however depend on sectoral mitigation actions, and thus should not suggest a relaxation of sectoral decarbonization targets.

## Abstract (web summary)

New study concludes that environmental tradeoffs of direct air capture and sequestration technologies are linked to the energy system in which they will operate, and their deployment should not equate to a relaxation of decarbonization or resource use efficiency targets.

## Introduction

Climate change mitigation scenarios used by the Intergovernmental Panel on Climate Change (IPCC)^1^ suggest that a rapid decarbonization in energy and material related services is likely to be insufficient to keep global mean temperature increase well below 2 °C by the end of the 21st century. The remaining global carbon budget of 420–1170 gigatonnes (Gt) CO_2_ is expected to be depleted in 10–30 years under present annual emission rates and projected Nationally Determined Contributions (NDCs)^2^. Most IPCC emission scenarios overshoot the carbon budget at first and then remove excess carbon via Carbon Dioxide Removal (CDR) technologies, i.e., intentional efforts to remove CO_2_ from the atmosphere and store it on land or in the oceans on the order of 200–1200 Gt CO_2_ toward the year 2100^2^.

CDR strategies include the enhancement of natural above- and belowground carbon sinks in plants, rock formations, and soils as well as scalable engineering solutions designed to sequester, store, or utilize concentrated atmospheric CO_2_. Direct Air Capture (DAC), despite being at an early stage of development, is gaining increasing attention and recognized as a promising climate change mitigation strategy^1^. Given the homogeneous atmospheric CO_2_ concentration levels around the world, DAC facilities can be deployed in locations that provide abundant cheap and carbon-free energy and/or that are close to pipeline infrastructure, underground storage, or utilization facilities for reducing the CO_2_ transportation cost^3^. Also, compared to bioenergy with carbon capture and storage (BECCS), an alternate CDR technology facilitating stringent mitigation targets^4^, DAC is expected to have much lower footprints in water and land uses^5^, reducing concerns around food security and biodiversity loss^6^.

Direct Air Carbon Capture and Storage (DACCS) uses chemical or physical processes to separate CO_2_ from ambient air and sequesters it permanently in geological storage sites. Due to the highly dilute nature of atmospheric CO_2_ (currently around 415 parts per million), DACCS technologies require substantial energy and material inputs, so their future deployment and role in climate change mitigation will depend heavily on process-design and resulting technoeconomic and environmental performances^3^. Two types of technologies are presently considered promising from a technoeconomic perspective: solvent-based DACCS, typically relying on aqueous hydroxide solutions (potassium hydroxide, sodium hydroxide) for capturing CO_2_^7–10^, and sorbent-based DACCS, mostly using amine materials bonded to a wide range of porous solid supports^11–14^. Solvent-based DACCS requires dedicated high-temperature (900 °C) heat for CO_2_ regeneration^10^. Thus, from a thermodynamic perspective, heat supply options are largely limited to combusting energy dense fuels such as (renewable) natural gas or (renewable) hydrogen, while electric resistance heating and electrochemical regeneration approaches are in development. Sorbent-based DACCS can function with low temperature (80–120 °C) heat for CO_2_ regeneration^15^, offering a larger variety of thermal energy supply options (e.g., heat pump, geothermal, and industrial waste heat).

A growing number of studies have included DACCS in integrated assessment modelling (IAM) scenarios. They highlight the critical role of DACCS in meeting stringent climate targets, but they also reveal the trade-offs of deploying DACCS, which, on the one hand, could reduce mitigation cost and relax the competition for land-use. On the other hand, large scale DACCS deployment and operation could also require large amounts of additional energy^16–19^. Depending on the modeling approach and scenario, these studies project that the DACCS deployment levels for meeting a 2 °C or stricter climate target by 2100 can reach up to 40 Gt of annual CO_2_ sequestration^16–18,20^. At this scale, DACCS (assuming a solvent-based process) could consume up to 12% and 60% global electric and non-electric energy by 2100^17,21^. Evidently, for DACCS facilities connected to electric power grids, their environmental performance will depend on the electricity system context in which they will operate. Previous studies have shown that DACCS can achieve negative emissions, but capture efficiencies are sensitive to the operational efficiency and the energy source^22–25^. A recent life cycle assessment (LCA) of DACCS technologies also identified potential environmental trade-offs in increased land transformation if DACCS is operated by solar electricity (as compared to using grid electricity)^26^. These studies, however, assume DACCS is powered either by a specific generation technology or static electricity systems. Thus, they neither reveal how environmental impacts of DACCS might change with energy system transitions following stringent mitigation scenarios^1^, nor do they quantify the potential broader environmental trade-offs of power system transitions with and without DACCS deployment in such scenarios toward 2100. Also, these studies do not fully account for long-term potential technological improvements of DACCS, which are expected to affect the environmental impacts of technologies by changing their physical material and energy inputs^27–29^.

Here, we calculate a prospective LCA of DACCS under climate change mitigation scenarios developed by the IMAGE 3.2 Integrated Assessment Model^30,31^ which are consistent with the climate targets of the Paris Agreement. IMAGE 3.2 has been used to project future energy supply, conversion, and demand toward 2100 across 26 global regions based on the demographic, economic, technological and behavioral narratives of the Shared Socioeconomic Pathways (SSPs)^32,33^. This study uses the ‘Middle of the Road’ pathway (SSP2), which assumes future developments in-line with historical patterns. This is then linked with climate targets defined by the Representative Concentration Pathways (RCPs)^34^ to determine required carbon prices which lead to changes in the energy system consistent with the achievement of specific climate targets. We use three distinct scenarios: An SSP2 baseline without any climate policies and measures to limit radiative forcing or to enhance adaptive capacity (SSP2-baseline). An SSP2 baseline linked with a strict climate change mitigation effort to limit global warming to less than 1.5 °C, i.e., a radiative forcing level of 1.9 W/m^2^ (RCP1.9), by 2100, allowing DACCS as a CDR option (SSP2-RCP1.9 w/ DACCS). Finally, a counterfactual that follows the same socioeconomic and climate change mitigation target but does not feature DACCS as a CDR option (SSP2-RCP1.9 w/o DACCS).

In an LCA study, the technological changes in both background and foreground systems can affect the environmental impacts of the studied object. The foreground system consists of processes directly related to the object, while the background system includes the upstream or downstream processes in the supply chain that are indirectly related to the object^35,36^. Here, we adapt an open-source LCA framework^37,38^ to modify electricity-related data in the background LCI database using regionally and temporally explicit IMAGE projections (on electricity mix, generation efficiency, and electricity-associated emissions) from 2020 to 2100 under the three scenarios. The regional impacts are differentiated for the United States (US) and compared to China, Russia, Western Europe, and a global average. Changes in the foreground material and energy inputs of the two technologies (solvent- and sorbent-based DACCS) over the same period are estimated based on the IMAGE projection of global DACCS deployment using a one-factor learning curve approach. We thus assume a commercial-scale operation and technology improvements via learning-by-doing. To capture the uncertainty related to the specific future learning rates, we apply different rates as part of a sensitivity analysis. Two types of heat supply options are also considered for solvent- (natural gas or biomethane) and sorbent-based DACCS (biomethane or heat pump) to understand how heat sources affect their environmental profiles. Furthermore, we also quantify the effect of DACCS deployment on the changes in power system loads, grid mixes, and related shifts in environmental impacts by comparing the strict mitigation scenario (SSP2-RCP1.9) with and without DACCS as a CDR option.

In this work, we find that decarbonizing the electricity sector improves the sequestration efficiency, but also increases the terrestrial ecotoxicity and metal depletion levels per tonne of CO_2_ sequestered via DACCS, but these increases can be reduced by improving the material and energy use efficiencies of DACCS under technology learning, indicating electricity sector decarbonization and DACCS technology improvements are both indispensable to avoid environmental problem-shifting. DACCS exhibits regional environmental impact variations, highlighting the importance of smart siting related to energy system planning and integration. DACCS deployment aids the achievement of long-term climate targets, its environmental and climate performances however depend on sectoral mitigation actions, and its deployment should not suggest a relaxation of sectoral decarbonization targets.

## Results

### Prospective life-cycle environmental impacts of DACCS in the US

**Fig. 1:**
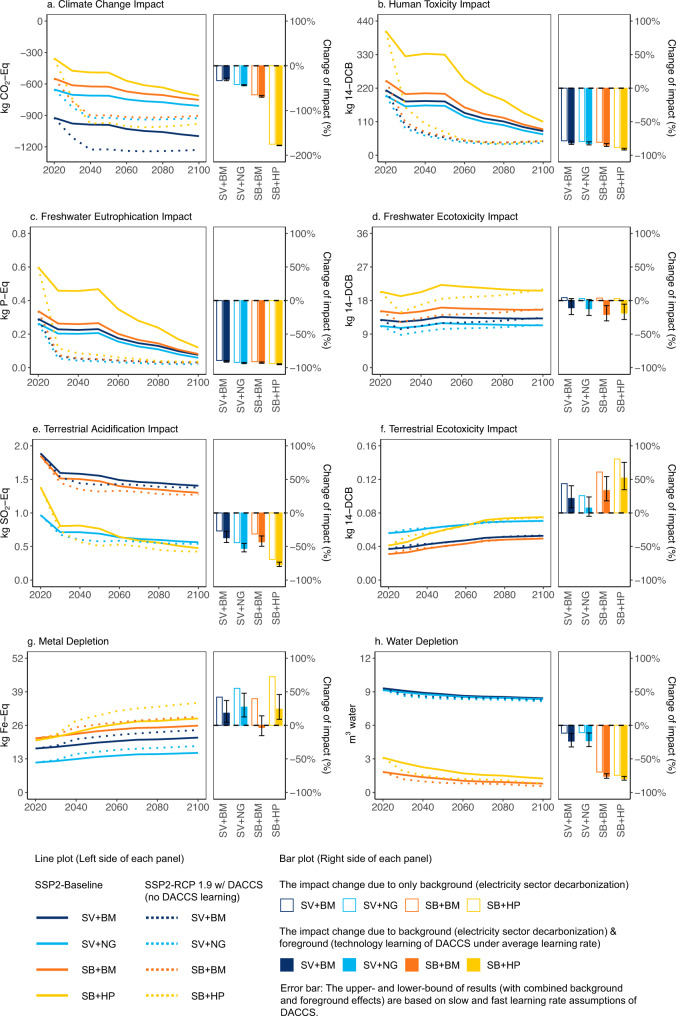
Prospective life cycle assessment results of direct air carbon capture and storage (DACCS) (per 1 t atmospheric CO_2_ captured and sequestered) from 2020 to 2100 considering background electricity sector decarbonization (United States (US) grid mix) and foreground technology learning of DACCS. Impact categories include (**a**) climate change impact, (**b**) human toxicity impact, (**c**) freshwater eutrophication impact, (**d**) freshwater ecotoxicity impact, (**e**) terrestrial acidification impact, (**f**) terrestrial ecotoxicity impact, (**g**) metal depletion, (**h**) water depletion. Four DACCS and heat source combinations are considered, including solvent-based DACCS with biomethane (SV + BM), solvent-based DACCS with natural gas (SV + NG), sorbent-based DACCS with biomethane (SB + BM), sorbent-based DACCS with heat pump (SB + HP). In each panel, the line plot (left side of each panel) shows the trajectory of environmental impacts due to the electricity sector decarbonization under two scenarios (excluding technological learning of DACCS). One is a “Shared Socioeconomic Pathways – Middle of the Road pathway” (SSP2) baseline scenario (SSP2-baseline). The second scenario links the SSP2 pathway with the Representative Concentration Pathway (RCP) that aligns with a radiative forcing level of 1.9 W/m^2^ (RCP1.9) by 2100 and allows DACCS as a CDR option (SSP2-RCP1.9 w/ DACCS). The bar plot (right side of each panel) includes technological learning of DACCS and thus compares the effects of the background and foreground systems (all under SSP2-RCP1.9 w/ DACCS scenario) on the environmental impacts of the four DACCS systems. The bars without color filling (only with boarder color) mark the percentage changes of impacts in 2100 relative to the 2020 level only due to the background electricity sector decarbonization, while the bars with color filling mark the percentage changes of impacts in 2100 relative to the 2020 level due to both background electricity sector decarbonization and foreground technology learning (based on reference learning rates) of DACCS. The error bars (associated to the bars with color filling) represent the results under slow and fast learning rates (Supplementary Table 10).

**Fig. 2:**
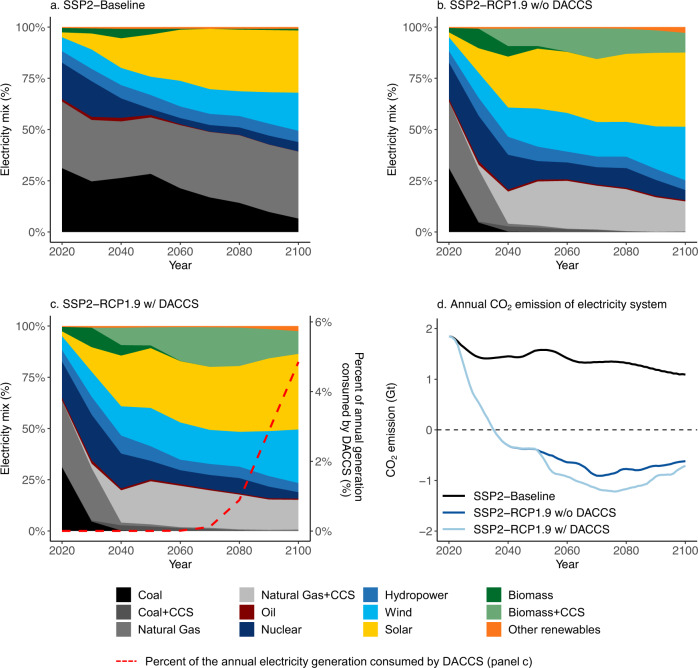
The relative technology contributions and CO_2_ emissions of the US electricity mix across different scenarios. The scenarios include (**a**) SSP2-baseline, (**b**) SSP2-RCP1.9 w/o DACCS (the SSP2 pathway linked with the RCP1.9 climate target, but does not include DACCS as a CDR option), (**c**) SSP2-RCP1.9 w/ DACCS scenarios and (**d**) the annual CO2 emissions of the US electricity system under the three scenarios. In the electricity mix panels (**a**, **b**, **c**), the stacked area represents the market shares of the grid mix. “Solar” includes both solar PV and concentrated solar power. “Oil” combines both oil with and without carbon capture and storage (CCS) as oil with CCS accounts for <1% of the grid mix. Other renewables include wave, tidal, and geothermal power. In (**c**), the red dashed line shows the percentage of the annual electricity generation consumed by DACCS, corresponding to the secondary *y*-axis.

DACCS achieves net negative greenhouse gas (GHG) emissions across all technologies and heat sources investigated per metric tonne (1t) of atmospheric CO_2_ captured and geologically sequestered in a US context by 2020. The net sequestration efficiency varies by DACCS technology and heat source (Fig. 1a) with life cycle climate change impacts ranging from −0.36 to −0.94t CO_2_-eq for a baseline grid-mix in 2020 (Fig. 1a). Net GHG negative implies that the DACCS technologies release less GHG emissions than they capture and geologically sequester over the plants’ life cycle (cradle-to-grave approach). The influence of different background electricity system contexts can be seen by comparing results for the SSP2-baseline vs. the SSP2-RCP1.9 w/ DACCS scenarios. In the SSP2-baseline, the US electricity system reduces the share of coal generation from 31% in 2020 to 7% in 2100, while its combined share of nuclear and renewable generation increases from 35 to 61% over the same period (Fig. 2a). As a result, the climate change impact of DACCS is further reduced to −0.72 to −1.12t CO_2_-eq by 2100. The highest sequestration efficiency is achieved by solvent-based DACCS using biomethane as a heat source (SV + BM). Since the process collects and sequesters CO_2_ released during the heat generation process step, using biomethane, a non-fossil, burden-free CO_2_ fuel, creates a negative CO_2_ emission profile beyond the 1t of atmospheric CO_2_ sequestered.

In the SSP2-RCP1.9 w/ DACCS scenario, the US electricity sector achieves a full decarbonization by 2035 (Fig, 2d), which is in-line with current targets and an economy-wide decarbonization by 2050^39^. The scenario features an earlier phase-out of coal and natural gas (by 2050) and higher renewable energy penetration (81%) by 2100 (Fig. 2c). In this scenario, the climate change impact of DACCS exhibits more rapid reductions before 2050 and reaches levels of −0.91 to −1.25 t CO_2_-eq by 2100 (Fig. 1a).

The life cycle human toxicity, freshwater eutrophication, terrestrial acidification, and water depletion of DACCS are sensitive to the shares of coal and natural gas generation in the electricity grid mix (Supplementary Fig. 9). These impacts decrease from 2020 to 2100, showing environmental co-benefits with decarbonizing the power sector (Fig. 1b, c, e, and h). Still, the US electricity system decarbonization creates environmental trade-offs for DACCS in other impact categories. We find increases for both terrestrial ecotoxicity (by 33–80% across four DACCS-heat source combinations for both SSP2-baseline and SSP2-RCP1.9 w/ DACCS scenarios) and metal depletion levels (by 23–42% and 40–73% across four DACCS-heat source combinations for SSP2-baseline and SSP2-RCP1.9 w/ DACCS scenario, respectively) from 2020 to 2100 given the growing contributions from solar photovoltaic (PV) and wind energy generation in the background electricity system (Fig. 2f, g, Supplementary Fig. 9). The increased ecotoxicity impact in scenarios with high renewable energy generation is largely due to emissions from the production of silicon-based solar PV cells and copper processing (as copper is used for wiring in solar PV and wind turbines). The higher relative metal demand (per kW installed) for the construction of solar PV and wind farms also increases mineral extraction. The electricity decarbonization barely affects the freshwater ecotoxicity of DACCS due to the counteracting effect of increased solar and wind penetrations (which raise the impact) and reduced coal generation (which decreases the impact) in the grid mix (Fig. 2d, Supplementary Fig. 9).

The life cycle environmental impacts of DACCS are affected by the technology type and heat source. The sorbent-based DACCS + heat pump (SB + HP) system has the highest climate change impact in 2020 because the heat is converted from fossil-dominated grid electricity, which has a higher carbon intensity than other heat supplies, but this impact is also more sensitive to electricity-sector decarbonization, so it shows a faster decrease over time. Under the SSP2-RCP1.9 w/ DACCS scenario, the climate change impact of the SB + HP system becomes the lowest compared to three other counterparts after 2040. For solvent-based DACCS, using biomethane as a heat source leads to a lower climate change impact than using natural gas due to the additional biogenic carbon sequestration. Hence, the SV + BM exhibits a lower life cycle climate change impact compared to the solvent-based DACCS system with natural gas (SV + NG) (Fig. 2a).

As for other non-climate metrics, sorbent-based DACCS generally exhibits higher impacts in human toxicity, freshwater eutrophication and ecotoxicity, and metal depletion mainly due to its higher unit electricity consumption. In contrast, solvent-based DACCS shows a higher water depletion (per 1 t CO_2_ captured, 3–12 times more than sorbent-based DACCS), because it captures CO_2_ using an aqueous hydroxide solution, which evaporates during the operation, while sorbent-based DACCS uses solid amine-based sorbents, which consumes much less water during the production and use phases. It has also been shown that, due to the affinity of amine sorbents for water, sorbent-based DACCS even co-produces water in humid environments, which can be used as freshwater or further purified into drinking water^15^. In terms of the heat source, solvent-based DACCS using natural gas heat has lower impacts for all studied categories compared to biomethane except for terrestrial ecotoxicity (higher impact due to the discarding of toxic drilling waste during natural gas production) and water depletion (which is more sensitive to the technology type than the heat source). Sorbent-based DACCS exhibits a lower environmental impact profile using biomethane for heat. The only increase compared to the heat pump derived heat is terrestrial acidification, which is mostly driven by the anaerobic digestion of biowaste in biomethane production (Fig. 2b–h).

Our results show that continuous improvements via learning-by-doing can mitigate some environmental impacts. Under the SSP2-RCP1.9 w/ DACCS scenario, technology learning starts to reduce material and energy inputs after 2050 when DACCS is deployed on a large-scale worldwide (Supplementary Table 11). Still, the climate change, human toxicity, and freshwater eutrophication impacts are mainly attributable to the electricity consumption (Supplementary Fig. 6) and the electricity sector decarbonization already decreases these impacts (of electricity generation) by more than 80% until 2050 (relative to 2020 levels) (Supplementary Fig. 9). Therefore, DACCS technology learning contributes <10% of the total changes (over the 80 years) in these impacts (Fig. 1a–c). While the electricity sector decarbonization increases freshwater ecotoxicity (slightly), terrestrial ecotoxicity, and metal depletion per tonne of CO_2_ sequestered via DACCS from 2020 to 2100, improvements in material and energy efficiency, induced by learning effects, have the potential to offset the increases across these categories. A sensitivity analysis confirms the prominent effect of learning in these impacts. Varying the learning rates between lower- and upper-bounds (Supplementary Table 10) causes additional increases (13–23%) or decreases (−10% to −13%) to the total changes of these impacts, while varying the learning rates barely affects the total impact changes for climate change, human toxicity, and freshwater eutrophication. Water depletion of solvent-based DACCS shows higher sensitivity to the change of learning rates compared to that of sorbent-based DACCS (Fig. 1h) as the solvent use accounts for more than 80% of the total water depletion for solvent-based DACCS (Supplementary Fig. 6). So, reducing the water evaporation during the operation can be an important strategy to decrease the life cycle water depletion of solvent-based DACCS.

### The impact of DACCS on the US electricity sector

**Fig. 3:**
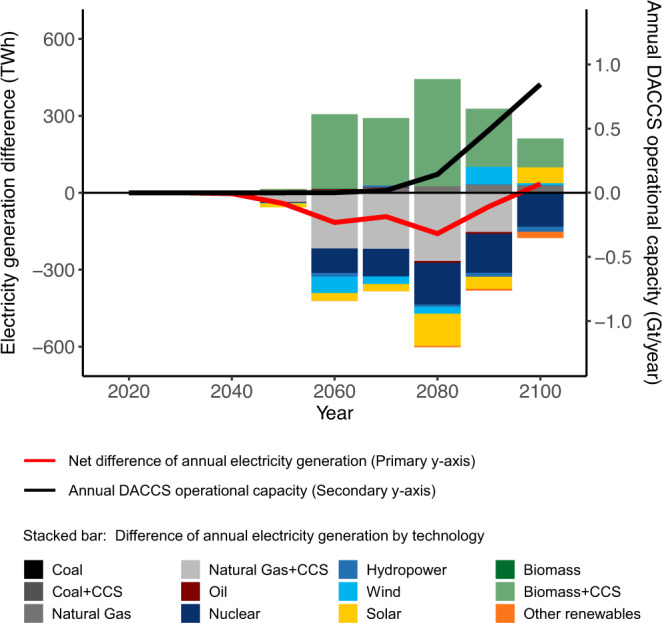
The change in US power generation with DACCS deployment. Stacked bars show the change of annual generation by technology when DACCS is a carbon dioxide removal option in the same mitigation scenario. The red line represents the net difference in annual power generation subtracting the SSP2-RCP1.9 w/o DACCS from the w/ DACCS scenario (primary *y*-axis). The black line represents the annual DACCS operational capacity (secondary *y*-axis).

The CDR capability provided by DACCS also affects the long-term development of the energy system. In our projections, carbon prices are used as a proxy to promote required changes in the energy system to limit emissions. Under the strict mitigation scenario with DACCS (SSP2-RCP1.9 w/ DACCS), DACCS deployment in the US starts around 2050, and its annual operational capacity reaches 0.85 GtCO_2_/year by 2100 (Fig. 3), consuming about 5% (352 TWh) of annual US electricity generation (Fig. 2c). The availability of DACCS essentially acts as a cap on the long-term carbon price, causing hard-to-abate sectors to offset their emissions using DACCS as opposed to investing in alternative technologies (e.g., electrification, energy efficiency improvement), and this leads to an increase in overall energy demand which is partially met by additional consumption of fossil fuel (natural gas, oil, and coal) (Supplementary Fig. 7a). Consequently, these hard-to-abate sectors promote additional CDR deployment, which is first met by additional CO_2_ sequestration from BECCS, which starts to increase after 2050, leading to an average 15% higher BECCS use as compared to the w/o DACCS scenario by 2080 (Supplementary Fig. 8a). Subsequently, as DACCS capacity increases more rapidly after 2080 and gradually meets the additional CDR demand, the annual CO_2_ sequestration from BECCS stabilizes around 1.3 GtCO_2_/year by 2100, like the levels in the strict mitigation scenario without DACCS. It is important to note, that on a global scale, the requirement of BECCS is lower in the SSP2-RCP1.9 w/ DACCS scenario than in the SSP2-RCP1.9 w/o DACCS case (Supplementary Fig. 8a).

The expansion of BECCS after 2050, peaking at 420 TWh/year by 2080, and reaching 113 TWh/year by 2100 is noticeable in the US generation mix when mapping out the differences between the two mitigation scenarios (Fig. 3). With DACCS, we also see that less electricity is generated from natural gas with carbon capture and storage (CCS) and nuclear during the same period, and the annual US electricity generation drops consistently during the BECCS expansion phase until 2080 (at −160 TWh/year or −2.3% compared to the without DACCS case). Thereafter, the rapid increase of DACCS operational capacity and the respective increase in electricity demand narrows the demand gap between the two scenarios. By 2100, 35 TWh/year of additional electricity are required under a mitigation scenario with DACCS.

**Fig. 4:**
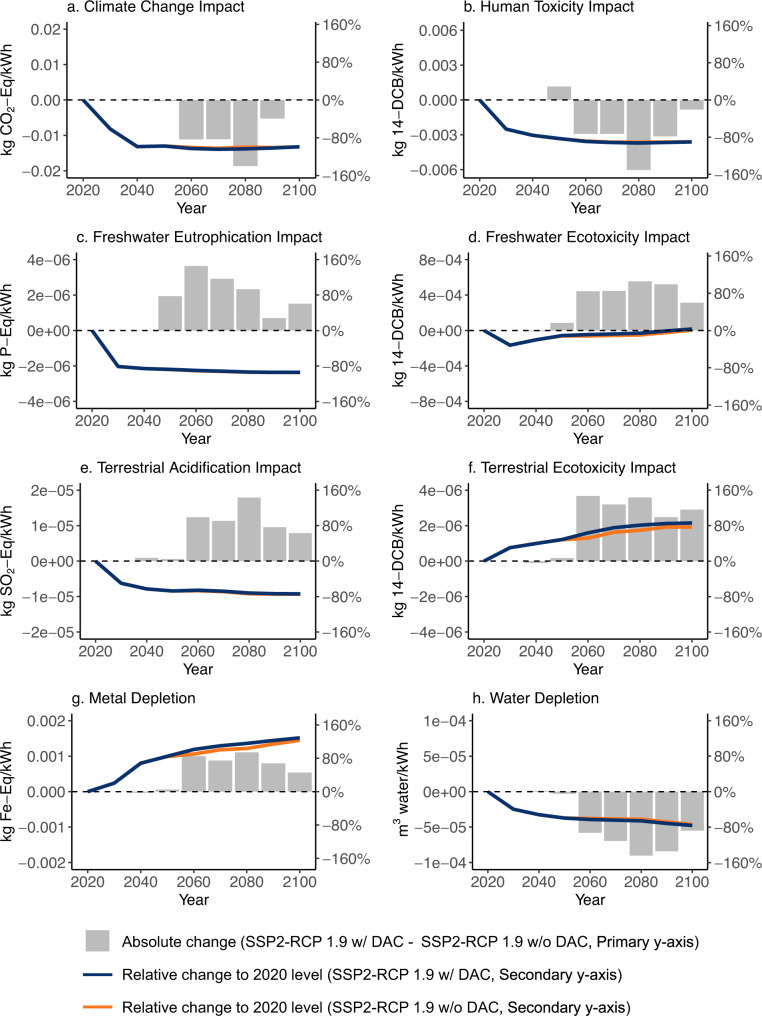
The change in life cycle impacts per unit (1 kWh) of US-based power generation with DACCS deployment. Impact categories include (**a**) climate change impact, (**b**) human toxicity impact, (**c**) freshwater eutrophication impact, (**d**) freshwater ecotoxicity impact, (**e**) terrestrial acidification impact, (**f**) terrestrial ecotoxicity impact, (**g**) metal depletion, (**h**) water depletion. The bar in each subplot represents the absolute change (per 1 kWh generation) of each impact subtracting the SSP2-RCP1.9 w/o DACCS from the w/ DACCS scenario from 2020 to 2100 (primary *y*-axis). The lines in each subplot represent the relative change (percentage) per impact compared to its 2020 reference level (secondary *y*-axis) under an RCP1.9 w/ (blue) and w/o DACCS scenario (orange).

The availability of DACCS barely changes the annual decarbonization rate of the US electricity system (about 6% across both scenarios based on the annual life cycle climate change impact). In both strict mitigation scenarios, the US power system reaches carbon neutrality by 2035 (Fig. 1d), which is in-line with the present US administration’s decarbonization target for the sector^39^. Beginning in 2050, the US grid mix starts to change with increasing DACCS deployment, leading to shifts in the long-term life cycle environmental impacts per kWh produced. We find a decrease in climate change impact up to −0.019 kg CO_2_-eq/kWh, which is mainly attributable to additional power generation from BECCS. Reductions also occur in water depletion and human toxicity impacts per kWh. At the same time, impacts of US power generation increase for several other categories including freshwater eutrophication and ecotoxicity, terrestrial acidification and ecotoxicity, and metal depletion (bars in Fig. 4). This environmental problem-shifting is directly attributable to the power grid mix change caused by DACCS. Still, for most impact categories, the changes are indiscernible compared to those caused by the electricity system decarbonization overall (lines in Fig. 4). Exceptions are metal depletion and terrestrial ecotoxicity, whose levels increase by 123% and 77% respectively from 2020 to 2100 due to the decarbonization of the power sector. DACCS deployment contributes an additional 10% (on average) after 2050 to both impact categories (Fig. 4).

### Environmental impacts of DACCS in other world regions

**Fig. 5:**
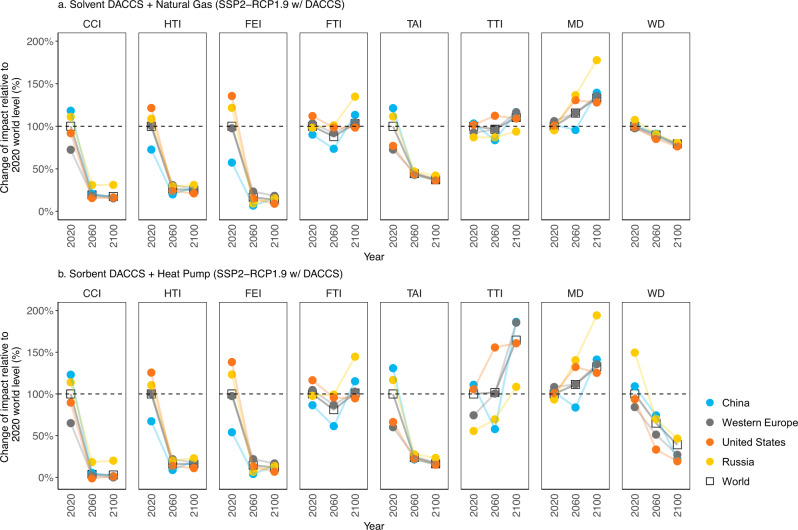
The regional variation of life cycle environmental impacts of DACCS technologies. Impacts of (**a**) solvent-based DACCS using natural gas (SV + NG) and (**b**) sorbent-based DACCS using heat pump generated heat (SB + HP) in four regions and the world under a SSP2-RCP1.9 w/ DACCS scenario (considering technology learning of DACCS with the reference learning rates). Per impact category, the reference (100% in 2020) is the World level. The results of other region-year combinations are shown as a relative change to the reference. These impact changes were calculated based on capturing and sequestering 1t atmospheric CO_2_ by DACCS. Since the technologies’ net negative life cycle Climate Change Impact (CCI) (in Fig. 1) would create a positive increase in impacts relative to the 2020 world level, we do not account for the 1t CO_2_ captured in the CCI in this figure. Other impact category abbreviations (from left to right): HTI Human Toxicity Impact, FEI Freshwater Eutrophication Impact, FTI Freshwater Ecotoxicity Impact, TAI Terrestrial Acidification Impact, TTI Terrestrial Ecotoxicity Impact, MD Metal Depletion, WD Water Depletion.

To put the US-specific results in a global context, we calculate the life cycle environmental impacts of DACCS using regionally explicit LCI data for electricity generation in China, Western Europe, and Russia as well as a global average under a SSP2-RCP1.9 w/ DACCS scenario (considering technology learning of DACCS). Since the solvent- and sorbent-based DACCS systems are commonly associated with thermal energy supply from natural gas (SV + NG) and heat pumps (SB + HP) respectively, these two configurations were considered representative processes for a global comparison. The results show that, in 2020, the climate change impact of SV + NG systems deployed in Russia and China are 12% and 19% higher than the same system at world level, because the electricity grid mixes in these regions are dominated by coal and natural gas, respectively (Fig. 5a, Supplementary Fig. 2, Supplementary Fig. 3). A higher climate change impact is also observed for SB + HP systems deployed in these two regions (14% and 23% for Russia and China, respectively) (Fig. 5b). Both DACCS systems exhibit lower climate change impacts than the 2020 world level if they are deployed in the US (9% and 10% less for SV + NG and SB + HP systems) and Western Europe (29% and 35% less for SV + NG and SB + HP systems) given the regions’ lower carbon-intensive electricity (Fig. 2, Supplementary Fig. 4). With time, the climate change impacts of DACCS decrease across all regions, and so do the regional variations. By 2100, climate change impacts barely differ across regions and the global average level, with slightly higher numbers observed for DACCS in Russia whose electricity mix is largely dominated by natural gas with CCS (33% of annual generation) (Supplementary Fig. 3). Similarly, decreasing trends of regional variations are observed for human toxicity, freshwater eutrophication, and terrestrial acidification impacts resulting from a worldwide decarbonization of the electricity sector under the mitigation scenario to limit global mean temperature change to below 1.5 °C by 2100. The ranges of regional variations remain stable for freshwater and terrestrial ecotoxicity and increase for metal depletion over time due to different renewable penetration levels and grid mix profiles across the regions. The water depletion of SB + HP systems is more sensitive to the regional electricity system context compared to that of SV + NG systems. Thus, SB + HP systems can reduce their already lower water demand even further with increasingly cleaner electricity toward 2100 (Fig. 5).

## Discussion

As more IAM scenarios start to include DACCS as a critical CDR technology for meeting stringent climate targets, the performance of DACCS should be evaluated in the context of those targets to better guide policy decision and deployment of DACCS in the future. As our LCA shows, a rapid decarbonization of the power and energy demand sectors that is consistent with the 1.5 °C climate target can increase the net sequestration efficiency of DACCS and facilitate its climate change mitigation potential, suggesting DACCS deployment and electricity system decarbonization should act synergistically in climate change mitigation efforts.

Several DACCS technologies can offset GHG emissions and aid with long-term climate change mitigation efforts, but their net sequestration efficiencies and holistic environmental performance are interdependent with the energy system in which they operate. Merely shifting to low-carbon energy sources for DACCS plant operation could lead to environmental trade-offs. These findings are in-line with other DACCS LCA studies^22,24,26^. We find that solvent-based DACCS generally has lower impacts than sorbent-based DACCS in five (climate change, human toxicity, freshwater eutrophication, freshwater ecotoxicity, and metal depletion) out of eight impact categories studied herein. This is contrary to the conclusions of another study, which states sorbent-based DACCS has lower environmental impacts for the impact categories considered therein (under the reference case)^40^. These differences appear to be linked to the study’s optimistic electricity (180 kWh/t CO_2_) and heat (2.6 GJ/t CO_2_) consumption assumptions for sorbent-based DACCS (under the reference case). These are less than half of those reported by several other studies^24,26,41^ and the ones used herein (470–700 kWh/t CO_2_ for electricity and 5.4–5.8 GJ/t CO_2_ for heat). Also, the study assumed that DACCS is powered by grid electricity in British Columbia, Canada, which is dominated by hydroelectricity (accounting for 72% of grid mix^42^) with low emissions for most impact categories. Thus, the environmental impacts (e.g., climate change, fossil depletion) of solvent-based DACCS were mainly driven by other factors such as a higher heat consumption. Furthermore, the study ignored the typical process-configuration for solvent-based DACCS in which the CO_2_ released during thermal energy generation^10^ is also captured and sequestered, thus artificially increasing the climate change impact of that technology and underestimating its potential sequestration efficiency. Neglecting this purposefully integrated process step not only alters the technology evaluation, it also leads to an underestimation of the storage capacity requirements and related inputs to regional planning and integration efforts. Solvent-based DACCS requires about 30% additional storage capacity (based on the 0.05 kg CO_2_/MJ^43^, which is the CO_2_ emission factor of natural gas combustion) per tonne of CO_2_ sequestered compared to sorbent-based DACCS.

Electricity consumption is a major contributor to the terrestrial ecotoxicity and metal depletion levels of DACCS, which are mainly driven by the solar and wind penetration levels in the background electricity system in our scenarios. Therefore, as the decarbonization of the electricity system progresses with expanding renewable energy generation and storage capacities, additional efforts are needed to facilitate sustainable mining, manufacturing, and expanding the circular economy of energy materials used in those technologies, which will reduce these impact levels.

Carbon management policies should consider research and development efforts to improve process and material efficiencies of DACCS and low-carbon energy generation technologies. DACCS technologies have already accomplished very high reuse rates of solvents and sorbents^10,24^, but our results show that technology learning prominently reduces levels of ecotoxicity, metal depletion, and water depletion (solvent-based DACCS only), highlighting its important role in avoiding potential environmental problem-shifting of DACCS deployment under a climate change mitigation pathway. Whereas large-scale DACCS deployment will affect the supply and demand dynamics of the overall energy system, this effect is negligible compared to the effects of decarbonizing the power sector. Thus, the deployment of DACCS is complementary to the expansion of other net-zero emission technologies as well as BECCS in stringent climate change mitigation scenarios.

Decarbonizing the electricity system substantially reduces regional differences of impacts, such as climate change, human toxicity, freshwater eutrophication, and terrestrial acidification, which are mostly driven by fossil-based energy generation. Still, varying environmental profiles across ecotoxicity and metal depletion persist toward 2100 under different renewable energy deployment strategies. This stresses the need for smart siting of DACCS, incorporating a wide range of environmental and socioeconomic metrics in the future to assess regional trade-offs. Given its load profile, DACCS deployment should also be integrated into regional energy system planning, including grid-connected and off-grid location assessments. DACCS could for instance be intentionally sited in locations with high renewable energy potential and where grid interconnections would be expensive.

The prospective LCA framework presented herein can inform policy discussions around research and development prioritization for emerging technologies that support energy sector decarbonization and long-term climate change mitigation targets. By incorporating regionally and temporally explicit electricity sector scenarios and technology projections for grid-connected DACCS, it captures the complex non-linear relationships between a CDR technology and its environmental impacts, caused by either changes in the broader energy system^44–46^ or its specific technology context^29,47^. Future capability extensions of this framework will model material circularity and capture the technological changes in broader energy and industrial sectors.

## Methods

### Overview

In this study, we adapt a cradle-to-grave LCA framework that evaluates temporally- and regionally-explicit environmental impacts of DACCS technologies in future electricity systems as projected by climate change mitigation scenarios^37^. The prospective framework aligns the temporal dimensions of the foreground technology learning and the background system dynamics. The life cycle impacts for the respective DACCS technologies are calculated using the Python-coded LCA framework Brightway2^48^ and life cycle inventory (LCI) data  from the ecoinvent database3.6^42^. The (background) electricity system context is provided by TIMER, the energy module of the IMAGE3.2 Integrated Assessment Model (IAM)^31^. TIMER develops regionally- and temporally-explicit projections for electricity mix, generation efficiency, and electricity-associated emissions, and these outputs are incorporated into another python-coded framework (Wurst)^37^ to update the electricity-related LCI data in the ecoinvent database, which is then used by Brightway2 to calculate the impacts per DACCS technology and time-step. The calculations are performed for 10-year timesteps from 2020 to 2100.

### Models

IMAGE 3.2 is an IAM framework developed to describe the relationships between humans and natural systems and the impacts of these relationships on the provision of ecosystem services to sustain human development^31^. The energy module of IMAGE 3.2, TIMER, is a recursive dynamic (i.e., no-foresight) energy system model representing the global energy system, disaggregated across 26 global regions, with projections till 2100^31^. It includes fossil and renewable primary energy carriers (coal, heavy/light oil, natural gas, modern/traditional biomass, nuclear, concentrated/PV solar, onshore/offshore wind, hydropower, and geothermal). Primary energy carriers can be converted to secondary and final energy carriers (solids, liquids, electricity, hydrogen, heat) to provide energy services for different end-use sectors (heavy industry, transport, residential, services, chemicals and other). The model projects future (useful) energy demand for each end-use sector (industry, transport, residential, commercial, other) based on relationships between energy services and activity, the latter of which is related to economic growth. For each demand sector, secondary energy carriers (including solid and liquid biofuels) compete based on relative costs with each other to meet the useful energy demand. The energy system representation of the IMAGE model does include demand elasticity with carbon prices. This is represented via two distinct mechanisms: (i) Investment in energy efficiency, and (ii) reduced demand in energy services (i.e., reducing consumption and foregoing activities and amenities which demand energy/emissions). The former is represented via technological options (i.e., invest in insulation, more efficient technologies, etc.) and the latter is represented based on econometric data. Energy prices are based on supply curves of energy carriers^49,50^. For non-renewable sources, these are formulated in terms of cumulative extraction; while for renewable sources, these are formulated in terms of annual production^51–53^.

Brightway2 is an open-source framework for LCA calculations in Python^48^. It consists of several modules that handle data import, managing and accessing data, calculating, and analyzing LCA results. The combination of a modular structure, the interactivity of Python, and tunable calculation pathways allows for flexibility and user-defined functionalities in conducting LCA studies and offers new possibilities compared to existing LCA tools.

Wurst is also a Python-based software that enables the systematic modification of LCI databases with external scenario data^37^. Wurst supports several generic modification types, including changing material efficiency, emissions, relative shares of markets inputs, and separating a global dataset into multiple regions. The current version of Wurst focuses on modifying the ecoinvent LCI database using IMAGE scenario data. More detailed information regarding modification steps of Wurst are discussed in the “LCI database modifications with climate scenario data” section.

### Scenario description

The Shared Socioeconomic Pathways – Middle of the Road baseline scenario (SSP2-baseline) projections assume no climate policy whatsoever, thus acting as a counterfactual to which policy efforts can be compared. The RCP1.9 scenarios project the required effort needed to meet a climate target, defined as an emission budget consistent with a 1.5 °C global mean temperature increase. These scenarios also include current climate policy, per region, as defined by the NDCs^54^. For the RCP1.9 scenarios, the IMAGE model determines the additional effort needed to meet the 1.5 °C target, represented by emission price projection across all GHG emission sources (fossil fuels, industry, and land use), applied globally, resulting in a cost-effective mitigation pathway. The emission price can reduce emissions via two mechanisms: (i) the increase in aggregate energy costs promotes investments in energy efficiency, (ii) by attaching this price to the carbon content of primary energy carriers, and it affects their competitiveness at meeting final energy demand services, thus promoting cleaner energy carriers. The application of an emission price makes DACCS competitive as it is assumed that sequestered carbon is renumerated, thus overcoming capital and variable costs (which in turn are affected by the projected cost of energy supply and technological learning). We present two RCP1.9 variations (SSP2-RCP1.9 w/ DACCS and SSP2-RCP1.9 w/o DACCS) to determine the impact of DACCS availability on climate change mitigation strategies. Regional cost-effectiveness in DACCS depends on capital and O&M costs (including endogenous learning-by doing reductions), electricity price, and CO_2_ transport and storage costs linked to storage potential limitations^55^. A single DACCS technology (with technology parameters and cost data based on plant capacity of 1 Mt CO_2_/year) is included in IMAGE, represented by aggregate of different solvent-based technologies summarized in previous studies^8,56,57^, but we assume that the DACCS deployment result estimated by IMAGE will represents the total deployment of a wide range of DACCS technologies (including both solvent- and sorbent-based DACCS). In IMAGE, it is assumed that DACCS is not available before 2030, and its global growth rate is limited to 1 GtCO_2_/year. This growth rate limit is a binding constraint in the projection once DACCS becomes cost effective, while in the long-term storage potential limitation may limit its further expansion. DACCS becomes cost effective when emission prices exceed approximately $300/tCO_2_. This emission price is surpassed in 2050 for both SSP2-RCP1.9 w/ DACCS and SSP2-RCP1.9 w/o DACCS. In the long-term, the application of DACCS limits the growth of the emission price, projected to be $423/tCO_2_ and $885/tCO_2_ 2100 for SSP2-RCP1.9 w/ DACCS and SSP2-RCP1.9 w/o DACCS respectively. By calculating the differences of electricity generation and the associated environmental impacts between the two RCP1.9 variations, we can also evaluate the effect of DACCS deployment on the electricity and energy demand systems.

### Technology assumptions and details of DACCS systems

We focus on two types of DACCS technologies: a solvent-based and a sorbent-based DACCS, which rely on different capture and release mechanisms to remove CO_2_ from the atmosphere.

Solvent-based DACCS applies aqueous hydroxide solutions (potassium hydroxide, sodium hydroxide) to capture atmospheric CO_2_ via a chemical reaction^7–10^. Here, we assume the solvent-based DACCS uses potassium hydroxide solution for CO_2_ capture. In an air contactor, the potassium hydroxide solution reacts with CO_2_ and forms potassium carbonate, which then, in a separate reactor, reacts with calcium hydroxide and generates calcium carbonate. The calcium carbonate precipitates, and potassium hydroxide solution can be regenerated and recycled back to the air contactor. The precipitated calcium carbonate is collected, dried, and then calcined under high temperature (about 900 °C) heat, which is typically provided by natural gas combustion in pure oxygen, to release the CO_2_. The CO_2_ released from calcium carbonate and the CO_2_ generated by natural gas combustion are mixed and collected for further storage^10^. The high temperature heat requirements limit the heat supply options for solvent-based DACCS. In this study, we consider natural and renewable gas (biomethane) as the two heat options for the solvent-based DACCS (Supplementary Fig. 1). Other proposed methods include electric resistance heating and electrochemical regeneration, which were not studied here.

Sorbent-based DACCS typically uses amine materials bonded to a wide range of porous solid supports for CO_2_ capture^11–14^. Here, we considered the use of amine-based silica as the solid sorbent^24^. The process consists of two main steps that operate cyclically: adsorption and desorption. In the adsorption step, a fan blows air through the air contactor, and the CO_2_ in the air reacts with the sorbent and binds to it. When the solid sorbent has been saturated with CO_2_, the desorption step will start in the air collector. Before heat is supplied, a vacuum is pulled to remove residual air from the contactor and decrease the temperature required for regeneration. Then, heat at about 100 °C will be supplied into the air contactor to desorb the CO_2_. The collected CO_2_ will then go through a cooling unit, where extra moisture can be removed through condensation and CO_2_ will be brought to ambient temperature. In the desorption step, the temperature of heat is about 80–120 °C, so a wide variety of thermal energy sources (natural gas, heat pump, geothermal heat, and waste heat) can be used as the heat supply. Here, we model heat pump (with coefficient of performance of 2.5^24^) and renewable gas (biomethane) as the two main options (Supplementary Fig. 1).

CO_2_ transport and storage: Once the CO_2_ is released from either process, we assume the CO_2_ flow will be compressed through a compressor to 11 MPa and then transported through a pipeline to the storage site. The length of the transport pipeline is assumed to be 50 km. At the storage site, the CO_2_ will be further compressed to 15 MPa and injected into a geological reservoir through wells with the depth of 3 km each. Here, the CO_2_ will be permanently stored as supercritical phase^58^(Supplementary Fig. 1).

### Life cycle assessment

The system boundary starts at the air inlet with a CO_2_ concentration of 415 ppm, and is followed by CO_2_ capture, regeneration, compression, transport, and ends with geological storage. Our analysis also accounts for upstream emissions due to indirect energy demands for the construction of energy conversion technologies, fuel production and handling. The functional unit is capturing and sequestering one metric tonne (1t) of atmospheric CO_2_ by DACCS technologies. The LCI data of the two studied DACCS technologies and subsequent compression and storage were collected from literature or estimated through bottoms-up materials requirements analysis (with the assumed plant capacities of 1 Mt CO_2_ and 0.1 Mt CO_2_ per year for solvent- and sorbent-based DACCS respectively), which are discussed in detail in Supplementary Note 1. The LCI data are assumed to represent the status quo material and energy consumptions over the life cycle of the two selected DACCS technologies. ReCiPe 2016 v1.1 hierarchist perspective is used as the characterization method to convert emissions and natural resource extractions to environmental impact categories at mid-point level^59^.

In this study, when we compare the environmental impacts of DACCS under different electricity decarbonization pathways (SSP2-baseline vs. SSP2-RCP1.9 w/ DACCS), the results are calculated based on static LCI data of DACCS that represent their current material and energy uses without considering technology learning. Then, we also calculated another set of LCA results for DACCS under SSP2-RCP1.9 w/ DACCS scenario based on dynamic LCI data that are estimated using learning curve approach, so it captures the effects of both background electricity decarbonization and foreground technology learning. By comparing the LCA results of DACCS calculated using static and dynamic LCI data under SSP2-RCP1.9 w/ DACCS scenario, we can evaluate and compare the effects of background electricity decarbonization and foreground technology learning on the environmental impacts of DACCS.

### Technology learning of DACCS systems

The learning curve approach has been used as an empirical method to study the unit cost reduction over time with cumulative production increases for a wide range of manufacturing^60^ and energy technologies^61^. The learning effect can be characterized by various mechanisms, including technology advancement, increased labor productivity, economies-of-scale, and improved material and energy efficiency. The learning curve approach has also been acknowledged as one critical means to explore the future expected life cycle impacts of present-day emerging technologies^62,63^. Here, we apply the one-factor learning curve approach to inform our prospective LCA. While the two technologies under investigation are presently operating in pilot- or demonstration scale, we assume a commercial-scale operation for both and apply constant learning rates, affecting the future life cycle material and energy consumption. Yet, for both technologies assessed herein, these learning effect on material and energy consumption are missing in the published literature. Thus, we assumed changes of material and energy consumption proportional to the changes per unit cost for the DACCS technologies.

It has been shown that the capital costs of solvent- and sorbent-based DACCS are likely to follow different learning rates given their different design characteristics. The solvent-based DACCS is site-built and large-scale, benefitting from economies-of-scale, but it is also less likely to incorporate rapid design or manufacturing improvement, while sorbent-based DACCS is based on standardized and modular units, and these units can be mass-produced and deployed, which enables fast iteration and learning^64^. Therefore, we assumed the average learning rates of 10% and 15% for the material and energy consumption that are related to capital investments for solvent- and sorbent-based DACCS, respectively. Then, as for the material and energy consumption related to operational costs, we assumed average learning rates of 2.5% for both solvent- and sorbent-based DACCS, respectively. We also consider variation ranges for the learning rates to reflect their uncertainty (Supplementary Table 10), these variation ranges are used to develop a sensitivity analysis to understand how the speed of learning affects the environmental impacts of DACCS. Furthermore, to avoid unrealistic reductions of material and energy consumption under technology learning, we also set up minimum material and energy use factors of both DACCS technologies based on expert estimations. As for the solvent-based DACCS, the lower bound of material and energy uses related to capital and operational costs cannot be lower than 44% and 50% of their original amounts, respectively, and the sorbent-based DACCS, the lower bound of material and energy uses related to capital and operational costs cannot be lower than 18% and 50% of their original amounts in 2020, respectively. To incorporate the minimum material and energy use factors into the learning curve formula, we adjusted the learning curve formula into the following Eq. 1:

1 $${D}_{i,t}={({D}_{i,0}-{D}_{i,{\min }})\times (1-{{LR}}_{i})}^{{{\log }}_{2}({X}_{t}/{X}_{0})}\,+\,{D}_{i,{\min }}$$

In Eq. 1, ${X}_{0}$ represents the initial DAC deployment capacity at year $0$; ${X}_{t}$ represents the cumulative DAC deployment capacity at year $t$. For a specific material or energy item $i$, ${{LR}}_{i}$ represents the learning rate of the item $i$; ${D}_{i,0}$ typically represents the unit consumption of the material or energy item $i$ at year $0$ (corresponding to the initial CO_2_ capture ${X}_{0}$). Here our goal is to calculate the material and energy use factors (instead of actual unit consumption) under technology learning, so we normalize the ${D}_{i,0}$ to be 1; ${D}_{i,t}$ is also a normalized material and energy use factors of item $i$ at year $t$ (corresponding to the cumulative CO_2_ capture ${X}_{t}$); ${D}_{i,{\min }}$ represents the minimum material and energy use factors of item $i$.

Finally, we assume that solvent- and sorbent-based DACCS each account for half of the global cumulative capacity of DACCS (IMAGE model outputs), respectively. Then, we estimated material and energy use factors for both solvent- and sorbent-based DACCS from 2020 to 2100 based on their cumulative capacity, and the results are presented in Supplementary Table 11. By multiplying the material and energy use factors at a specific year to the actual unit material and energy consumption at the initial year, we can get the actual unit material and energy consumption in that specific year. Assumptions on technology learning rates and minimum material and energy use factors of solvent- and sorbent-based DACCS are discussed in detail in Supplementary Note 2.

### LCI database modifications with climate scenario data

The ecoinvent database^23^ is the most widely used LCI database which offers fully interlinked unit process supply chains for products presented in the database. It covers all relevant environmental flows, material and energy inputs, and products of around 18,000 activities, where researchers can collect data about the supply chain to form a comprehensive background system in an LCA study. However, since the data in ecoinvent are usually collected in a specific year, the database describes the material and energy flows among processes based on an existing supply chain system. Therefore, the ecoinvent database is limited in conducting prospective LCA studies, which assess the environmental impacts associated to future technologies or emerging technologies that evolve over time.

Here, to evaluate the environmental impacts of DACCS technologies in a context of a changing background electricity system, we adapt an open-source approach (Wurst)^37^ that systematically integrates the IMAGE projections on electricity mix, generation efficiency, and electricity-associated emissions with the ecoinvent database, and change the parameters in electricity-related activity data in the ecoinvent database. Due to the differences of generation technologies between IMAGE and ecoinvent database, we develop a matching list to map the available technologies for both data sources (Supplementary Note 3). More detailed information regarding parameter modification for ecoinvent database using Wurst can be found in a previous study^37^. After the parameter modification, we developed 27 versions of ecoinvent databases, which correspond to 9 different years from 2020 to 2100 under the SSP2-baseline, SSP2-RCP1.9 w/ DACCS, and SSP2-RCP1.9 w/o DACCS scenarios.

### Limitations

In this study, we modify the background LCI database using IMAGE projections of grid mix, generation efficiency and emissions of thermal power plants (fossil-based sources, biomass, and nuclear), while the renewable sources and their efficiency levels are based on existing available technologies. Technological innovation has been observed for renewable (especially solar^65,66^ and wind^67^) and energy storage^68,69^ technologies, and they will continue to evolve as they are more widely applied in the energy system. Therefore, to better evaluate the prospective environmental impacts of energy-intensive technologies, such as DACCS, under specific climate contexts, the analysis framework could be expanded to consider the advancement, particularly in material efficiency or circularity of variable renewable energy and storage technologies in the background electricity system.

Previous studies looking at the technology learning of DACCS have focused on cost reductions^64,70,71^. Publicly available, empirical studies that reveal how material and energy inputs change as DACCS scales could not be identified. Given this limited data availability, we assume the material and energy inputs of DACCS follow the same learning rates as the associated cost projections. In reality, technology learning rates are likely to vary depending on processes and physical input types^29,72^. Future LCA studies aiming to quantify the effects of technology learning on environmental impacts might be able to rely on more detailed learning data of specific physical inputs. In addition, learning rates of emerging technologies tend to change with technology-readiness-levels (TRL)^73–75^. Prospective analyses of emerging technologies ideally reflect this by applying a multi-factor learning curve approach, differentiating between the varying learning rates at different TRL. The technologies analyzed herein operate at demonstration scale (TRL-7) while we apply a single-factor, constant learning rate, postulating learning-by-doing improvements at commercial scale (TRL-9). The learning rate at commercial scale is a research frontier and presently unknown. Yet, at the scale of our analysis, a respective differentiation is unlikely to add accuracy or insight. The uncertainty with respect to the specific single learning rate at commercial scale is captured by testing how different learning rates affect our results. Using a single-factor learning curve approach, we thus attribute the cost change and its related material and energy consumption to the cumulative installed capacity of DACCS over time, limiting our capability of revealing the correlation between technology progress and other factors, such as (prior) R&D expenditure^76^.

The life cycle impact assessment step relates emissions and resource use to environmental impacts through characterization factors. The framework we adapted here applied global or European scale characterization factors. While location-generic characterization factors are suitable for global impacts such as climate change impact, they may lead to large uncertainty for quantifying non-global impacts, such as acidification^77^, eutrophication^78^, and ecotoxicity^79^, which are typically affected by regional meteorological, hydrological, soil conditions and the sensitivity of ecosystems to emissions. While country-dependent characterization models and factors have been developed for these impact categories, they have not yet been incorporated into the LCA framework applied in this study. Further methodological improvements are needed to enhance the capability of the existing framework for conducting regional impact assessments.

This study shows the environmental impacts of DACCS could have different trajectories depending on the background energy system, so it is important to keep monitoring those environmental metrics or even considering them in the decision-making process. Future research could explore the feasibility of incorporating life cycle environmental metrics into IAMs for better environmental impact assessment. State-of-the art IAMs typically include some environment-related metrics, such as GHG emission, land and water use as constraints, but they lack many other environmental impact dimensions. For example, metal consumption could be an important metric given the increasing penetration of renewable and battery storage in the energy system, which are resource intensive. Furthermore, life cycle environmental metrics capture the emissions from all life cycle phases (e.g., construction, transport, operation, and end-of-life, etc.), and IAMs evaluate the interrelationship among different sectors. Therefore, the integration of life cycle environmental metrics and IAMs should carefully allocate the emissions of different life cycle phases to the corresponding sectors/energy carriers in IAM to avoid double counting^80^.

## Supplementary information

Supporting Information

Description of Additional Supplementary Information

Supplementary Data 1

## Data Availability

The complete LCI data for the two DACCS technologies are provided in the Supplementary Information document (Table 6 to Table 9). The IMAGE outputs of the three scenarios considered in this study are documented and provided in the “Data_source” file in the Supplementary Dataset. The LCI data of some electricity generation technologies (wave, fossil fuel with CCS), biomethane heat supply, and amine-base sorbent are incorporated into the modified ecoinvent database during the LCA modeling process, these LCI data are also provided in the same “Data_source” file in the Supplementary Dataset. A permanent reference of our data provided in GitHub repository is also accessible through 10.5281/zenodo.6513343^81^.

## Code availability

All the R and Python codes used in this study are documented and provided in the “Code” file in the Supplementary Dataset. R Programming is used to make the figures in this study, and all the results used to make figures are documented and provided in the “R_code_result_export” file in the Supplementary Dataset. To rerun the LCA model calculations, Brightway2, Wurst (two python coding frameworks), and the Ecoinvent 3.6 database (license required) are needed. A permanent reference of our code provided in GitHub repository is also accessible through 10.5281/zenodo.6513343^81^.

## References

[1] IPCC. *Special report on the impacts of global warming of 1.5* *°C above pre-industrial levels and related global greenhouse gas emission pathways*. (Intergovernmental Panel on Climate Change, 2018).

[2] Rogelj, J. et al. Scenarios towards limiting global mean temperature increase below 1.5 °C. *Nat. Clim. Change* **8**, 325–332 (2018).

[3] Sanz-Pérez ES, Murdock CR, Didas SA, Jones CW (2016). Direct Capture of CO2 from Ambient Air. Chem. Rev..

[4] Bauer N (2020). Global energy sector emission reductions and bioenergy use: overview of the bioenergy demand phase of the EMF-33 model comparison. Clim. Change.

[5] Smith P (2016). Biophysical and economic limits to negative CO2 emissions. Nat. Clim. Change.

[6] Fuss S (2018). Negative emissions—Part 2: Costs, potentials and side effects. Environ. Res. Lett..

[7] Stucki S, Schuler A, Constantinescu M (1995). Coupled CO2 recovery from the atmosphere and water electrolysis: Feasibility of a new process for hydrogen storage. Int. J. Hydrog. Energy.

[8] Baciocchi R, Storti G, Mazzotti M (2006). Process design and energy requirements for the capture of carbon dioxide from air. Chem. Eng. Process. Process Intensif..

[9] Zeman F (2007). Energy and Material Balance of CO2 Capture from Ambient Air. Environ. Sci. Technol..

[10] Keith DW, Holmes G, St. Angelo D, Heidel K (2018). A Process for Capturing CO2 from the Atmosphere. Joule.

[11] Veselovskaya JV (2013). Direct CO2 capture from ambient air using K2CO3/Al2O3 composite sorbent. Int. J. Greenh. Gas. Control.

[12] Lu W, Sculley JP, Yuan D, Krishna R, Zhou H-C (2013). Carbon dioxide capture from air using amine-grafted porous polymer networks. J. Phys. Chem. C..

[13] Gebald C, Wurzbacher JA, Tingaut P, Zimmermann T, Steinfeld A (2011). Amine-Based Nanofibrillated Cellulose As Adsorbent for CO _2_ Capture from Air. Environ. Sci. Technol..

[14] McDonald TM (2012). Capture of carbon dioxide from air and flue gas in the alkylamine-appended metal–organic framework mmen-Mg2 (dobpdc). J. Am. Chem. Soc..

[15] Beuttler C, Charles L, Wurzbacher J (2019). The Role of Direct Air Capture in Mitigation of Anthropogenic Greenhouse Gas Emissions. Front. Clim..

[16] Chen C, Tavoni M (2013). Direct air capture of CO2 and climate stabilization: a model based assessment. Clim. Change.

[17] Marcucci A, Kypreos S, Panos E (2017). The road to achieving the long-term Paris targets: energy transition and the role of direct air capture. Clim. Change.

[18] Fuhrman J (2020). Food–energy–water implications of negative emissions technologies in a +1.5 °C future. Nat. Clim. Change.

[19] Realmonte G (2019). An inter-model assessment of the role of direct air capture in deep mitigation pathways. Nat. Commun..

[20] Fuhrman, J. et al. The role of direct air capture and negative emissions technologies in the shared socioeconomic pathways towards $\mathplus$1.5 °C and $\mathplus$2 °C futures. *Environ. Res. Lett.* **16**, 114012 (2021).

[21] Creutzig F (2019). The mutual dependence of negative emission technologies and energy systems. Energy Environ. Sci..

[22] de Jonge MMJ, Daemen J, Loriaux JM, Steinmann ZJN, Huijbregts MAJ (2019). Life cycle carbon efficiency of Direct Air Capture systems with strong hydroxide sorbents. Int. J. Greenh. Gas. Control.

[23] Liu CM, Sandhu NK, McCoy ST, Bergerson JA (2020). A life cycle assessment of greenhouse gas emissions from direct air capture and Fischer–Tropsch fuel production. Sustainable. Energy Fuels.

[24] Deutz S, Bardow A (2021). Life-cycle assessment of an industrial direct air capture process based on temperature–vacuum swing adsorption. Nat. Energy.

[25] McQueen, N., Desmond, M. J., Socolow, R. H., Psarras, P. & Wilcox, J. Natural Gas vs. Electricity for Solvent-Based Direct Air Capture. *Front. Clim.* **2**, 618644 (2021).

[26] Terlouw, T., Treyer, K., Bauer, C. & Mazzotti, M. Life Cycle Assessment of Direct Air Carbon Capture and Storage with Low-Carbon Energy Sources. *Environ. Sci. Technol.* **55**, 11397–11411 (2021). 10.1021/acs.est.1c03263 34351133

[27] Caduff M, Huijbregts MAJ, Althaus H-J, Hendriks AJ (2011). Power-Law Relationships for Estimating Mass, Fuel Consumption and Costs of Energy Conversion Equipments. Environ. Sci. Technol..

[28] Caduff M, Huijbregts MAJ, Koehler A, Althaus H-J, Hellweg S (2014). Scaling Relationships in Life Cycle Assessment. J. Ind. Ecol..

[29] Bergesen JD, Suh S (2016). A framework for technological learning in the supply chain: A case study on CdTe photovoltaics. Appl. Energy.

[30] Van Vuuren DP (2017). Energy, land-use and greenhouse gas emissions trajectories under a green growth paradigm. Glob. Environ. Change.

[31] Stehfest, E., van Vuuren, D., Bouwman, L. & Kram, T. *Integrated assessment of global environmental change with IMAGE 3.0: Model description and policy applications*. (Netherlands Environmental Assessment Agency (PBL), 2014).

[32] Riahi K (2017). The shared socioeconomic pathways and their energy, land use, and greenhouse gas emissions implications: an overview. Glob. Environ. Change.

[33] O’Neill BC (2017). The roads ahead: Narratives for shared socioeconomic pathways describing world futures in the 21st century. Glob. Environ. Change.

[34] Van Vuuren DP (2011). The representative concentration pathways: an overview. Clim. change.

[35] Tillman A-M (2000). Significance of decision-making for LCA methodology. Environ. Impact Assess. Rev..

[36] Hospido A, Davis J, Berlin J, Sonesson U (2010). A review of methodological issues affecting LCA of novel food products. Int J. Life Cycle Assess..

[37] Beltran AM (2020). When the Background Matters: using Scenarios from Integrated Assessment Models in Prospective Life Cycle Assessment. J. Ind. Ecol..

[38] Rauner S (2020). Coal-exit health and environmental damage reductions outweigh economic impacts. Nat. Clim. Change.

[39] FACT SHEET: President Biden Sets 2030 Greenhouse Gas Pollution Reduction Target Aimed at Creating Good-Paying Union Jobs and Securing U.S. Leadership on Clean Energy Technologies. https://www.whitehouse.gov/briefing-room/statements-releases/2021/04/22/fact-sheet-president-biden-sets-2030-greenhouse-gas-pollution-reduction-target-aimed-at-creating-good-paying-union-jobs-and-securing-u-s-leadership-on-clean-energy-technologies/ (2021).

[40] Madhu K, Pauliuk S, Dhathri S, Creutzig F (2021). Understanding environmental trade-offs and resource demand of direct air capture technologies through comparative life-cycle assessment. Nat. Energy.

[41] McQueen N (2020). Cost Analysis of Direct Air Capture and Sequestration Coupled to Low-Carbon Thermal Energy in the United States. Environ. Sci. Technol..

[42] Wernet G (2016). The ecoinvent database version 3 (part I): overview and methodology. Int. J. Life Cycle Assess..

[43] Carbon Dioxide Emissions Coefficients by Fuel -U.S. Energy Information Administration (EIA). https://www.eia.gov/environment/emissions/co2_vol_mass.php (2021).

[44] Kätelhön A, Bardow A, Suh S (2016). Stochastic Technology Choice Model for Consequential Life Cycle Assessment. Environ. Sci. Technol..

[45] Kätelhön A, von der Assen N, Suh S, Jung J, Bardow A (2015). Industry-Cost-Curve Approach for Modeling the Environmental Impact of Introducing New Technologies in Life Cycle Assessment. Environ. Sci. Technol..

[46] Qin, Y., Yang, Y., Cucurachi, S. & Suh, S. Non-linearity in marginal LCA: application of spatial optimization model. *Front. Sustain.* **2**, 631080 (2021).

[47] Pizzol, M., Sacchi, R., Köhler, S. & Anderson Erjavec, A. Non-linearity in the Life Cycle Assessment of Scalable and Emerging Technologies. *Front. Sustain.* **1**, 611593 (2021).

[48] Mutel C (2017). Brightway: an open source framework for life cycle assessment. J. Open Source Softw..

[49] Mulders, F. M. M., Hettelar, J. M. M. & Van Bergen, F. Assessment of the global fossil fuel reserves and resources for TIMER*.* TNO Built Environment and Geosciences, Utrecht **98**, https://models.pbl.nl/image/index.php/Mulders_et_al.,_2006 (2006).

[50] Rogner H-H (1997). An assessment of world hydrocarbon resources. Annu. Rev. Energy Environ..

[51] De Vries BJ, Van Vuuren DP, Hoogwijk MM (2007). Renewable energy sources: Their global potential for the first-half of the 21st century at a global level: an integrated approach. Energy policy.

[52] Gernaat DE, Bogaart PW, van Vuuren DP, Biemans H, Niessink R (2017). High-resolution assessment of global technical and economic hydropower potential. Nat. Energy.

[53] Daioglou V, Doelman JC, Wicke B, Faaij A, van Vuuren DP (2019). Integrated assessment of biomass supply and demand in climate change mitigation scenarios. Glob. Environ. Change.

[54] van Soest, H. et al. A Global Roll-out of Nationally Relevant Policies Bridges the Emissions Gap. 10.21203/rs.3.rs-126777/v1 (2021).

[55] Koelbl BS, van den Broek MA, Faaij APC, van Vuuren DP (2014). Uncertainty in Carbon Capture and Storage (CCS) deployment projections: a cross-model comparison exercise. Clim. Change.

[56] Broehm, M., Strefler, J. & Bauer, N. Techno-Economic Review of Direct Air Capture Systems for Large Scale Mitigation of Atmospheric CO2. https://papers.ssrn.com/abstract=2665702. 10.2139/ssrn.2665702. (2015)

[57] Socolow, R. et al. Direct air capture of CO_2_ with chemicals: a technology assessment for the APS Panel on Public Affairs. https://www.aps.org/policy/reports/assessments/upload/dac2011.pdf. (2011).

[58] Koornneef J, van Keulen T, Faaij A, Turkenburg W (2008). Life cycle assessment of a pulverized coal power plant with post-combustion capture, transport and storage of CO2. Int. J. Greenh. Gas. Control.

[59] Huijbregts MA (2017). ReCiPe2016: a harmonised life cycle impact assessment method at midpoint and endpoint level. Int. J. Life Cycle Assess..

[60] Argote L, Epple D (1990). Learning Curves in Manufacturing. Science.

[61] Junginger, M. & Louwen, A. *Technological Learning in the Transition to a Low-Carbon Energy System: Conceptual Issues, Empirical Findings, and Use, in Energy Modeling*. (Academic Press, 2019).

[62] van der Giesen, C., Cucurachi, S., Guinée, J., Kramer, G. J. & Tukker, A. A critical view on the current application of LCA for new technologies and recommendations for improved practice. *J. Cleaner Product.* **259**, 120904. 10.1016/j.jclepro.2020.120904. (2020).

[63] Bergerson JA (2020). Life cycle assessment of emerging technologies: evaluation techniques at different stages of market and technical maturity. J. Ind. Ecol..

[64] Baker, S. E. et al. Getting to neutral: options for negative carbon emissions in California. https://gs.llnl.gov/sites/gs/files/2021-08/getting_to_neutral.pdf. (2019).

[65] Ahmad L, Khordehgah N, Malinauskaite J, Jouhara H (2020). Recent advances and applications of solar photovoltaics and thermal technologies. Energy.

[66] Rabaia MKH (2021). Environmental impacts of solar energy systems: a review. Sci. Total Environ..

[67] Watson S (2019). Future emerging technologies in the wind power sector: a European perspective. Renew. Sustain. Energy Rev..

[68] Aneke M, Wang M (2016). Energy storage technologies and real life applications – A state of the art review. Appl. Energy.

[69] Schmidt O, Hawkes A, Gambhir A, Staffell I (2017). The future cost of electrical energy storage based on experience rates. Nat. Energy.

[70] Fasihi M, Efimova O, Breyer C (2019). Techno-economic assessment of CO2 direct air capture plants. J. Clean. Prod..

[71] McQueen N (2021). A review of direct air capture (DAC): scaling up commercial technologies and innovating for the future. Prog. Energy.

[72] Karali N, Park WY, McNeil M (2017). Modeling technological change and its impact on energy savings in the U.S. iron and steel sector. Appl. Energy.

[73] Gross, R. et al. *Presenting the future: electricity generation cost estimation Methodologies*. https://d2e1qxpsswcpgz.cloudfront.net/uploads/2020/03/presenting-the-future-electricity-generation-cost-estimation-methodologies.pdf. (UK Energy Research Centre, 2013).

[74] Ferioli, F. & Van der Zwaan, B. C. C. *Learning in times of change: A dynamic explanation for technological progress*. (ACS Publications, 2009). 10.1021/es900254m 19569322

[75] Nemet GF (2009). Interim monitoring of cost dynamics for publicly supported energy technologies. Energy Policy.

[76] Rubin ES, Azevedo IML, Jaramillo P, Yeh S (2015). A review of learning rates for electricity supply technologies. Energy Policy.

[77] Roy P-O (2014). Characterization factors for terrestrial acidification at the global scale: a systematic analysis of spatial variability and uncertainty. Sci. Total Environ..

[78] Helmes RJK, Huijbregts MAJ, Henderson AD, Jolliet O (2012). Spatially explicit fate factors of phosphorous emissions to freshwater at the global scale. Int J. Life Cycle Assess..

[79] Dong Y, Rosenbaum RK, Hauschild MZ (2016). Assessment of Metal Toxicity in Marine Ecosystems: Comparative Toxicity Potentials for Nine Cationic Metals in Coastal Seawater. Environ. Sci. Technol..

[80] Arvesen A, Luderer G, Pehl M, Bodirsky BL, Hertwich EG (2018). Deriving life cycle assessment coefficients for application in integrated assessment modelling. Environ. Model. Softw..

[81] Qiu, Y. Environmental trade-offs of direct air capture technologies in climate change mitigation toward 2100 — code and data. 10.5281/zenodo.6513343 (2022). 10.1038/s41467-022-31146-1 PMC9233692 35752628

