# Supplementary material for: Environmental trade-offs of direct air capture technologies in climate change mitigation toward 2100
Source: Nat Commun. 2022 Jun 25;13:3635. doi: 10.1038/s41467-022-31146-1 (PMC9233692; doi:10.1038/s41467-022-31146-1)
Supplement: Supplementary file 1 — Supporting Information [file 41467_2022_31146_MOESM1_ESM.pdf]

Supplementary Information

Environmental trade-offs of direct air capture technologies in climate change mitigation toward 2100

Yang Qiu[1,2], Patrick Lamers[1*], Vassilis Daioglou[3,4], Noah McQueen[5], Harmen-Sytze de Boer[4], Mathijs Harmsen[3,4], Jennifer Wilcox[5], André Bardow[6,7], Sangwon Suh[2*]

[1] National Renewable Energy Laboratory, 15013 Denver W Pkwy, Golden, CO 80401, United States
[2] Bren School of Environmental Science and Management, 2400 University of California, Santa Barbara, California, United States, 93117.
[3] Copernicus Institute of Sustainable Development, Utrecht University, Princetonlaan 8a, 3584 CS Utrecht, the Netherlands.
[4] PBL Netherlands Environmental Assessment Agency, PO Box 30314, 2500 GH The Hague, the Netherlands
[5] Chemical and Biomolecular Engineering Department, University of Pennsylvania, Philadelphia, PA, United States of America, 19104
[6] Institute of Energy and Climate Research - Energy Systems Engineering (IEK-10), Forschungszentrum Jülich GmbH, Jülich, Germany
[7] Energy and Process Systems Engineering, ETH Zurich, 8092 Zurich, Switzerland
* Correspondence: patrick.lamers@nrel.gov, suh@bren.ucsb.edu

Supplementary Figure 1

a. Solvent-based direct air carbon capture and storage (DACCS) system

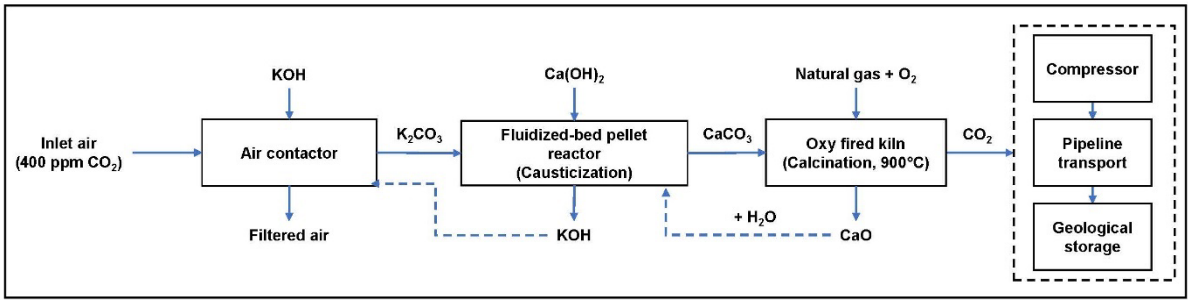

b. Sorbent-based DACCS system

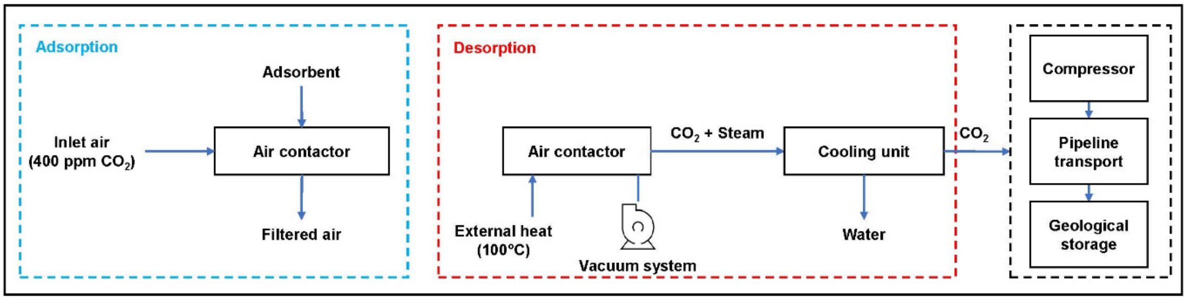

Supplementary Figure 1 | The process flow diagrams of solvent-based DACCS system (a) and sorbent-based DACCS system (b) with subsequent compression and storage system.

Supplementary Figure 2

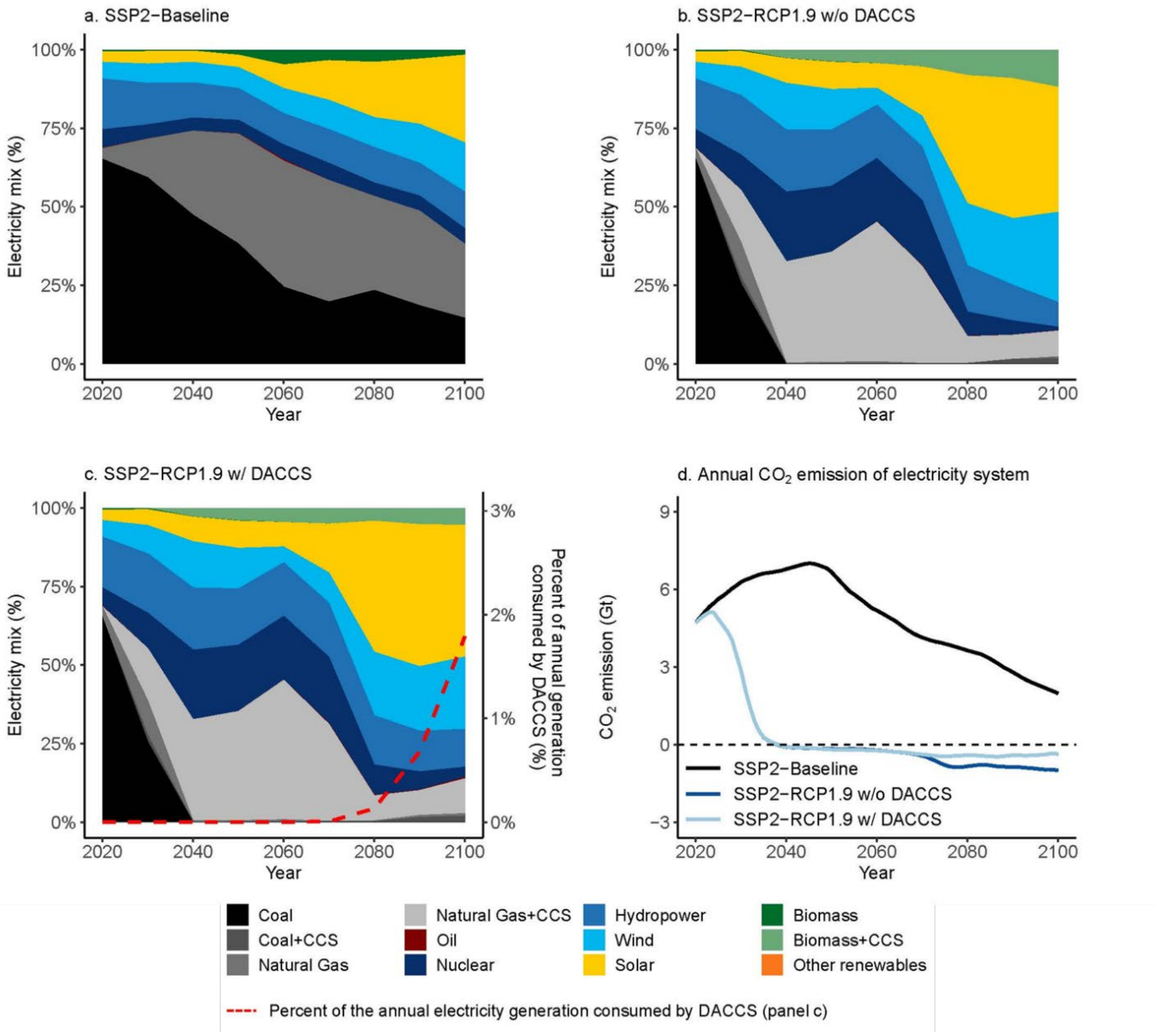

Supplementary Figure 2 | The relative technology contributions and CO_2 emissions of China's electricity mix across different scenarios. The scenarios include China's electricity mix under (**a**) SSP2-baseline, (**b**) SSP2-RCP1.9 w/o DACCS, (**c**) SSP2-RCP1.9 w/ DACCS scenarios and (**d**) the annual CO_2 emissions of the China's electricity system under the three scenarios. In the electricity mix panels (**a, b, c**), the stacked area represents the market shares of the grid mix. "Solar" includes both solar PV and concentrated solar power. "Oil" combines both oil with and without carbon capture and storage (CCS) as oil with CCS accounts for <1% of the grid mix. Other renewables include wave, tidal, and geothermal power. In (**c**), the red dashed line shows the percentage of the annual electricity generation consumed by DACCS, corresponding to the secondary y-axis.

Supplementary Figure 3

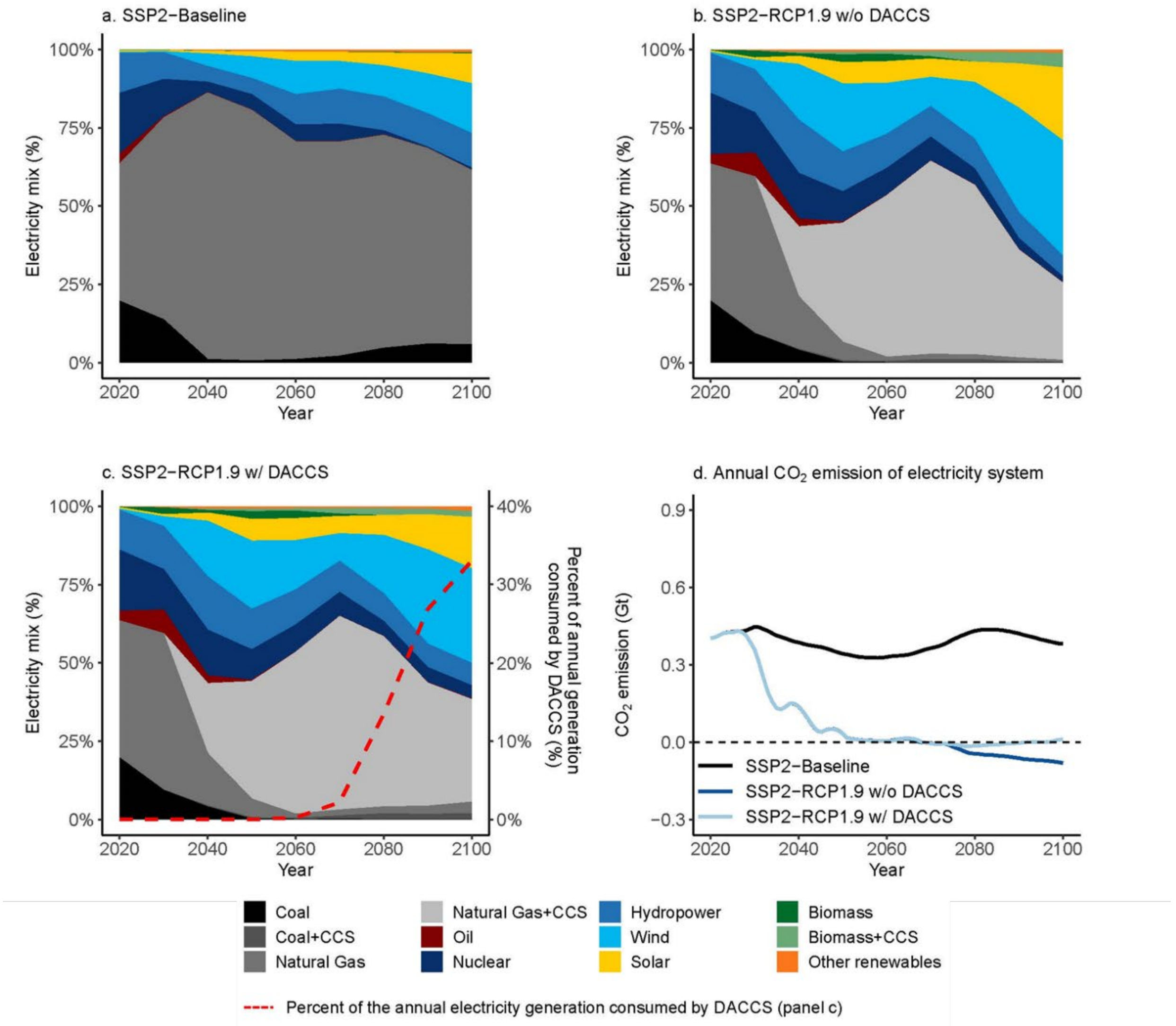

Supplementary Figure 3 | The relative technology contributions and CO_2 emissions of Russia's electricity mix across different scenarios. The scenarios include Russia's electricity mix under (**a**) SSP2-baseline, (**b**) SSP2-RCP1.9 w/o DACCS, (**c**) SSP2-RCP1.9 w/ DACCS scenarios and (**d**) the annual CO_2 emissions of the Russia's electricity system under the three scenarios. In the electricity mix panels (**a, b, c**), the stacked area represents the market shares of the grid mix. "Solar" includes both solar PV and concentrated solar power. "Oil" combines both oil with and without carbon capture and storage (CCS) as oil with CCS accounts for <1% of the grid mix. Other renewables include wave, tidal, and geothermal power. In (**c**), the red dashed line shows the percentage of the annual electricity generation consumed by DACCS, corresponding to the secondary y-axis.

Supplementary Figure 4

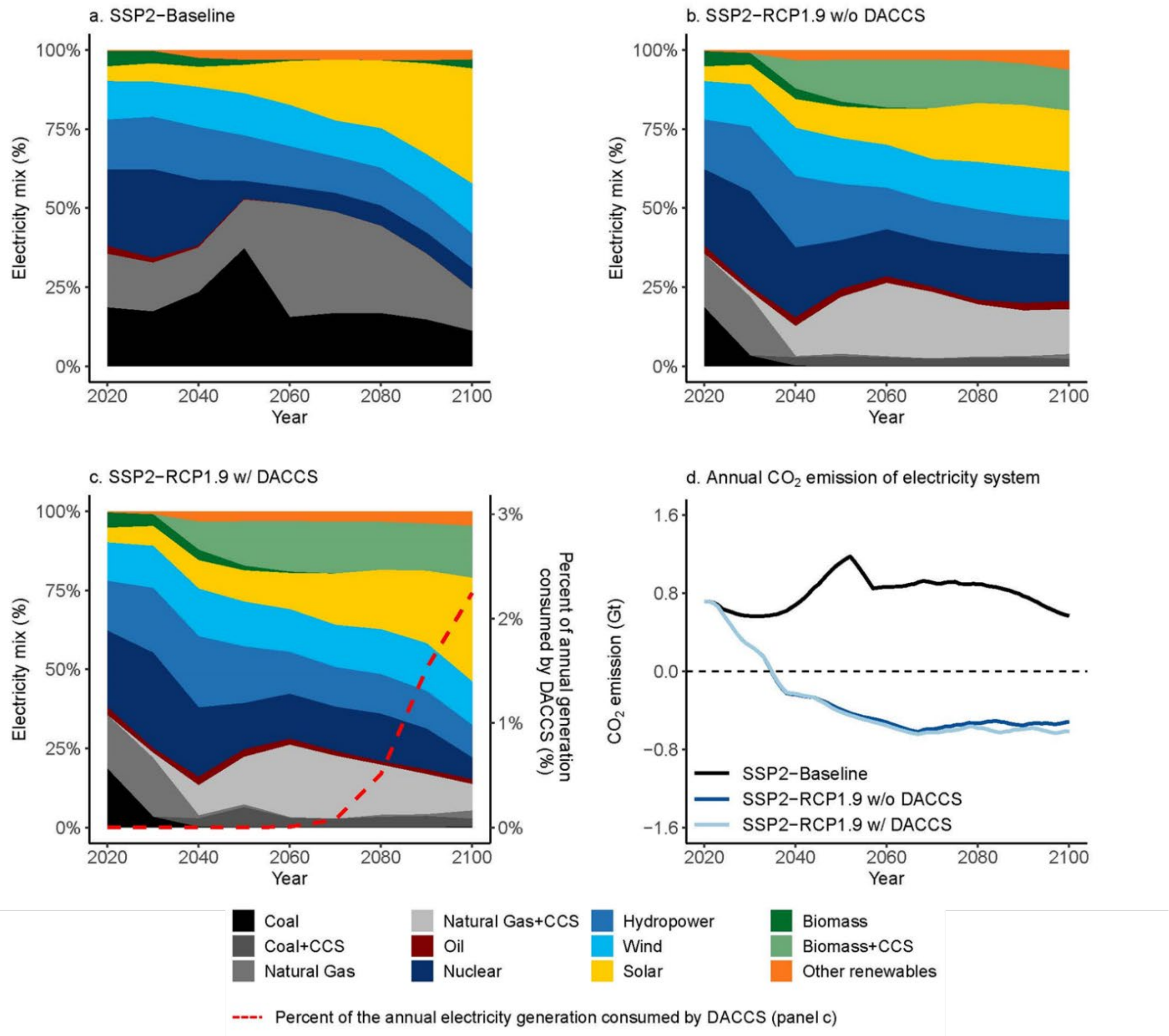

Supplementary Figure 4 | The relative technology contributions and CO_2 emissions of the Western European electricity mix across different scenarios. The scenarios include Western European electricity mix under (**a**) SSP2-baseline, (**b**) SSP2-RCP1.9 w/o DACCS, (**c**) SSP2-RCP1.9 w/ DACCS scenarios and (**d**) the annual CO_2 emissions of the Western European electricity system under the three scenarios. In the electricity mix panels (**a, b, c**), the stacked area represents the market shares of the grid mix. “Solar” includes both solar PV and concentrated solar power. “Oil” combines both oil with and without carbon capture and storage (CCS) as oil with CCS accounts for <1% of the grid mix. Other renewables include wave, tidal, and geothermal power. In (**c**), the red dashed line shows the percentage of the annual electricity generation consumed by DACCS, corresponding to the secondary y-axis.

Supplementary Figure 5

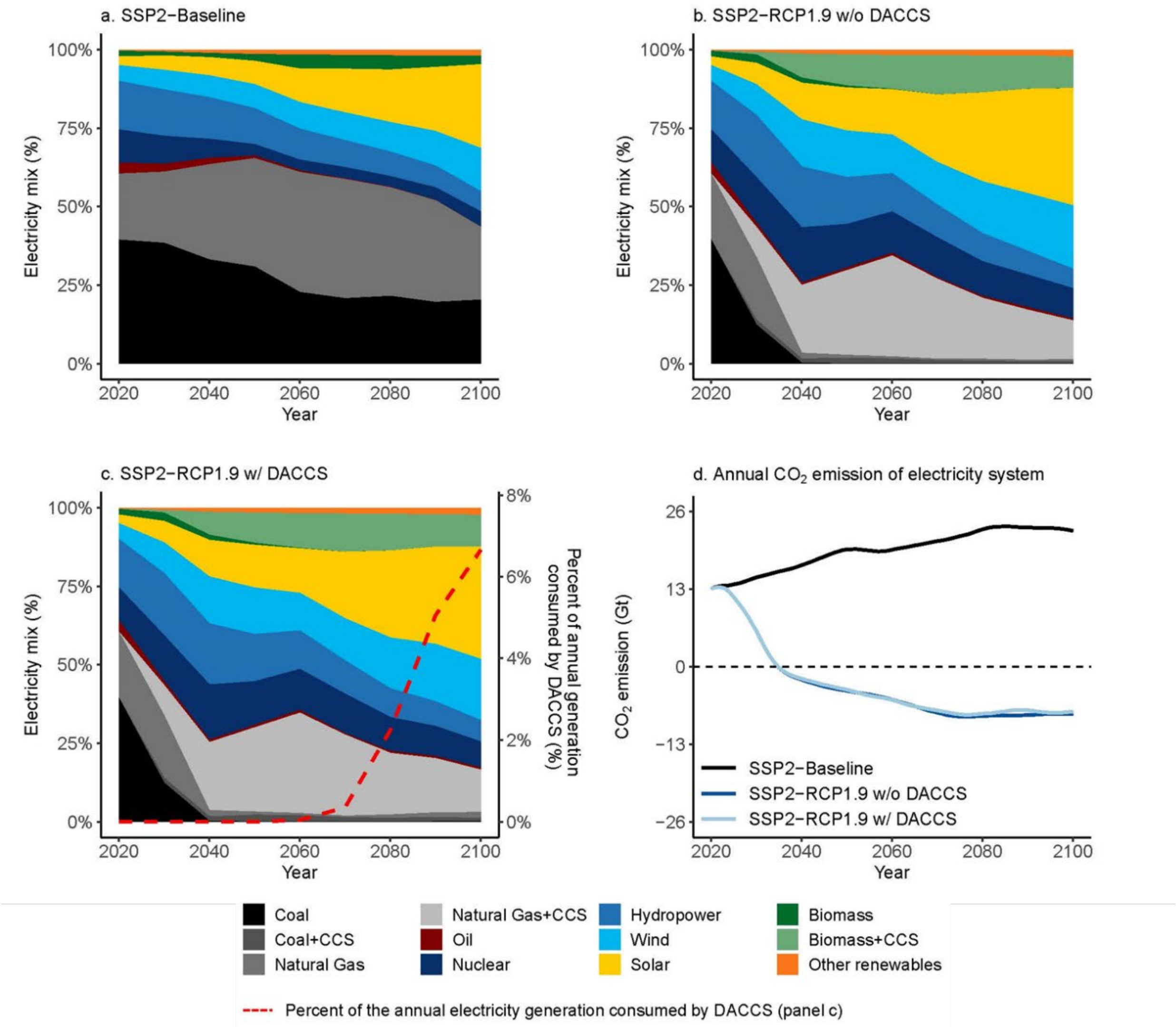

Supplementary Figure 5 | The relative technology contributions and CO_2 emissions of the world electricity mix across different scenarios. The scenarios include world electricity mix under (**a**) SSP2-baseline, (**b**) SSP2-RCP1.9 w/o DACCS, (**c**) SSP2-RCP1.9 w/ DACCS scenarios and (**d**) the annual CO_2 emissions of the world electricity system under the three scenarios. In the electricity mix panels (**a, b, c**), the stacked area represents the market shares of the grid mix. "Solar" includes both solar PV and concentrated solar power. "Oil" combines both oil with and without carbon capture and storage (CCS) as oil with CCS accounts for <1% of the grid mix. Other renewables include wave, tidal, and geothermal power. In (**c**), the red dashed line shows the percentage of the annual electricity generation consumed by DACCS, corresponding to the secondary y-axis.

Supplementary Figure 6

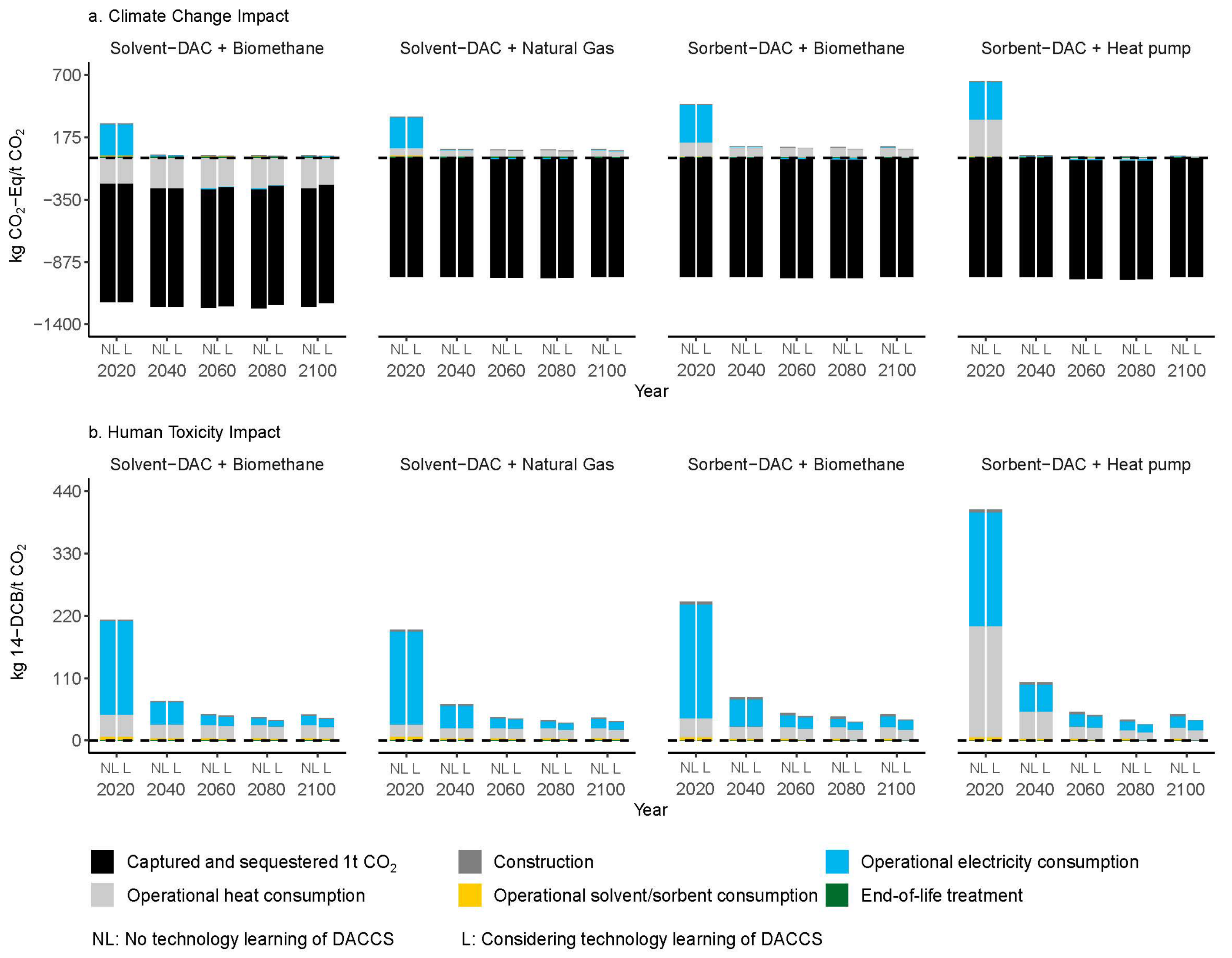

Figure to be continued

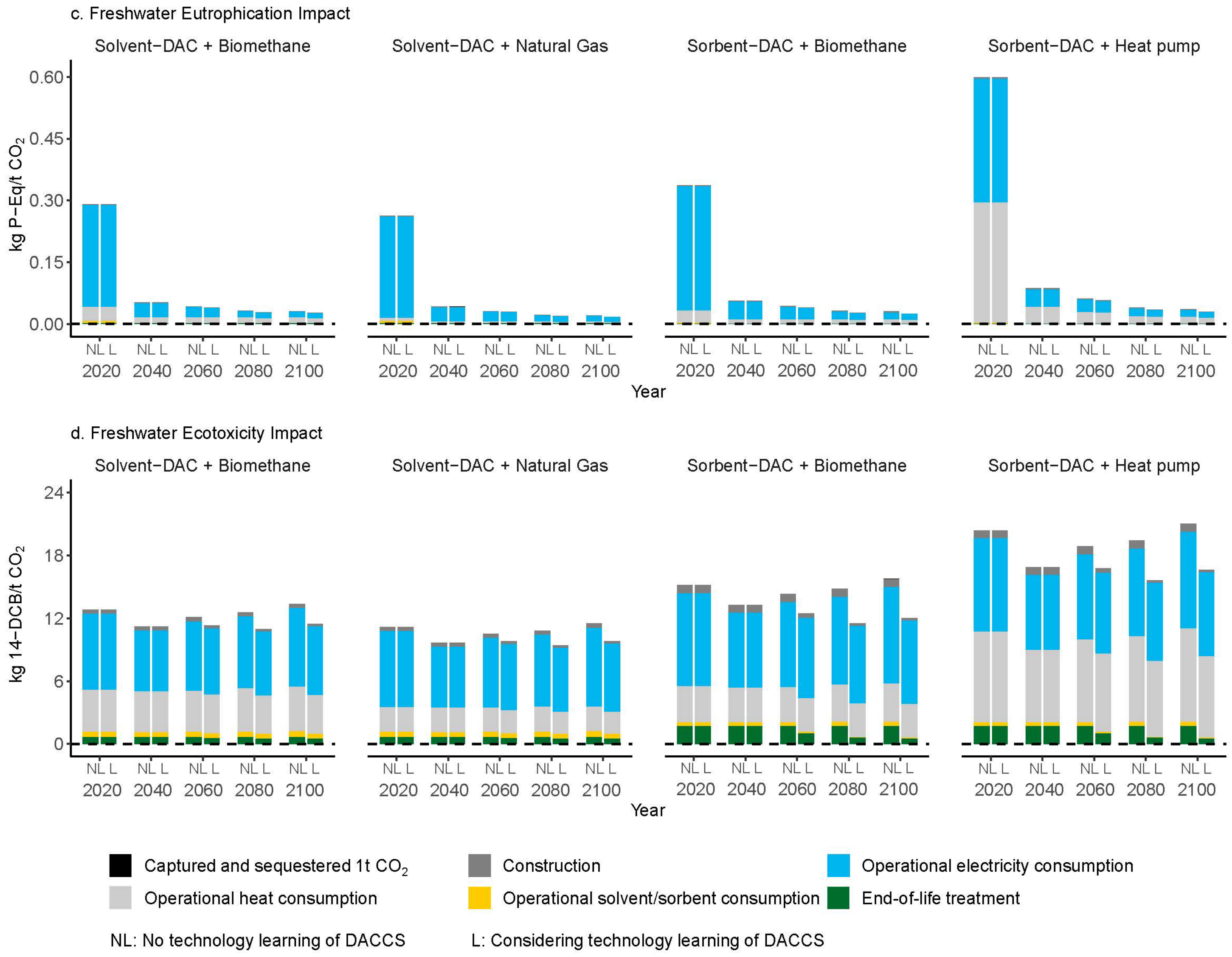

Figure to be continued

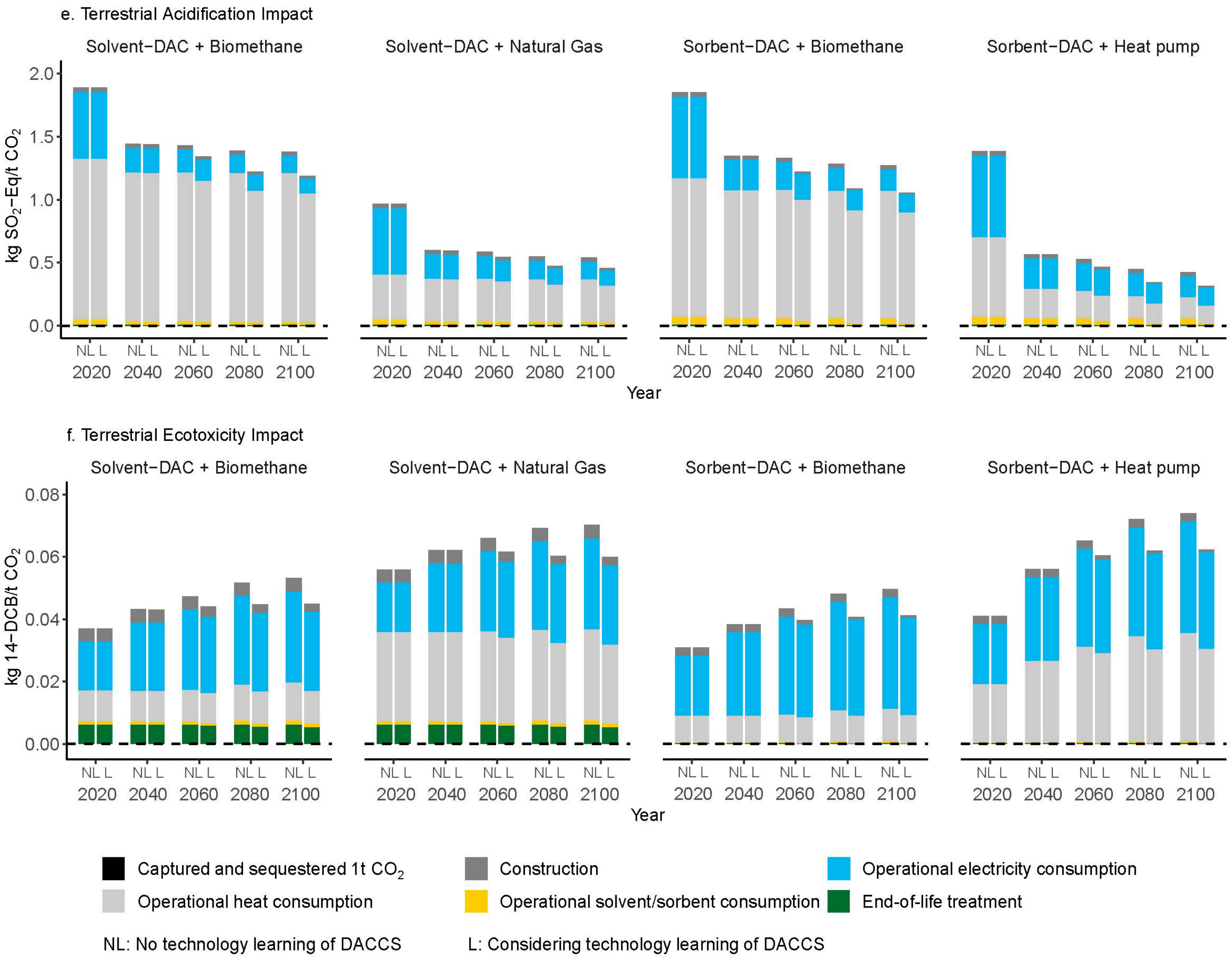

Figure to be continued

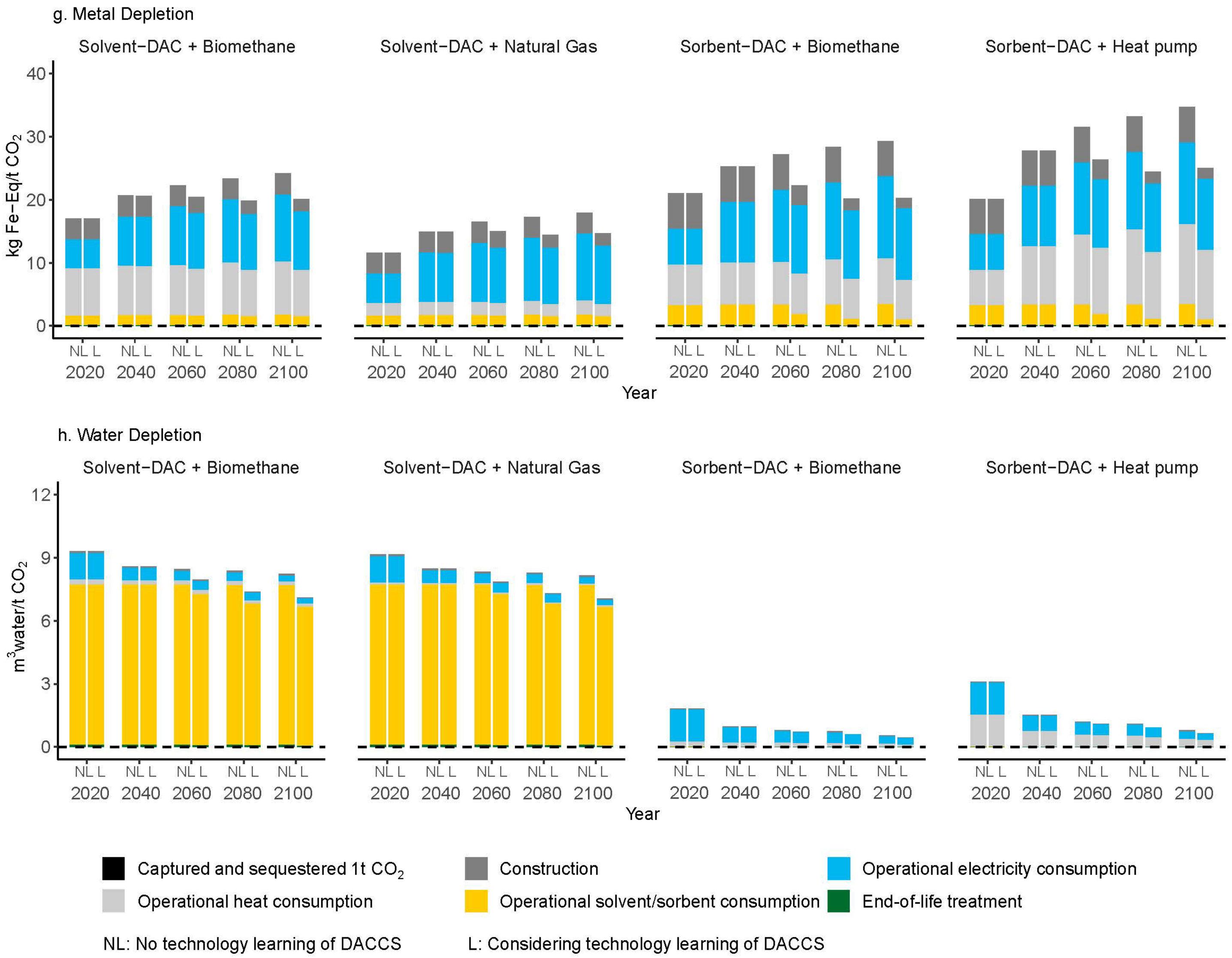

Supplementary Figure 6 | The contribution of different life cycle stages to the total environmental impact (per 1 t atmospheric CO_2 captured and sequestered) under SSP2-RCP1.9 w/DAC scenario (US case). Impact categories include (a) climate change impact, (b) human toxicity impact, (c) freshwater eutrophication impact, (d) freshwater ecotoxicity impact, (e) terrestrial acidification impact, (f) terrestrial

ecotoxicity impact, (g) metal depletion, (h) water depletion. Under each impact category, the results include four DACCS and heat sources combinations (Solvent-based DACCS that uses biomethane or natural gas as a heat source. Sorbent-based DACCS that uses biomethane or heat pump as a heat source). The colors of the stacked bar represent different life cycle stages. Each year corresponds to two bars representing the results of no technology learning (“NL”, on the left) and with technology learning (“L”, on the right).

Supplementary Figure 7

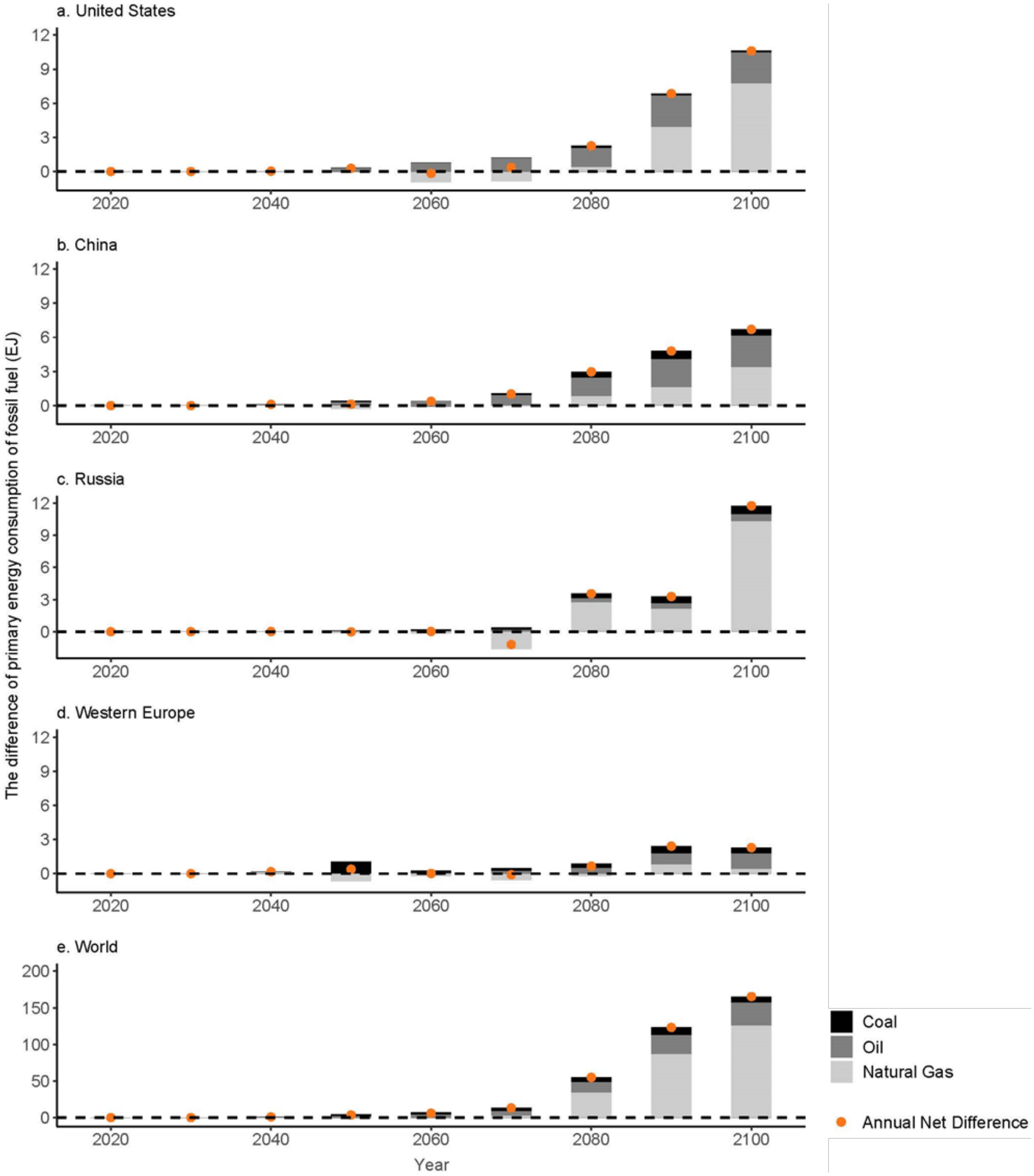

Supplementary Figure 7 | The difference of annual total primary energy consumption of fossil fuels (coal, oil and natural gas) in the United States (a), China (b), Russia (c), Western Europe (d) and the World as a whole (e) between SSP2-RCP1.9 w/o DACCS and SSP2-RCP1.9 w/ DACCS scenarios. The difference is calculated by subtracting the primary energy consumption under SSP2-RCP1.9 w/o DACCS scenario from that under SSP2-RCP1.9 w/ DACCS scenario. The red dots represent the net difference in each year.

Supplementary Figure 8

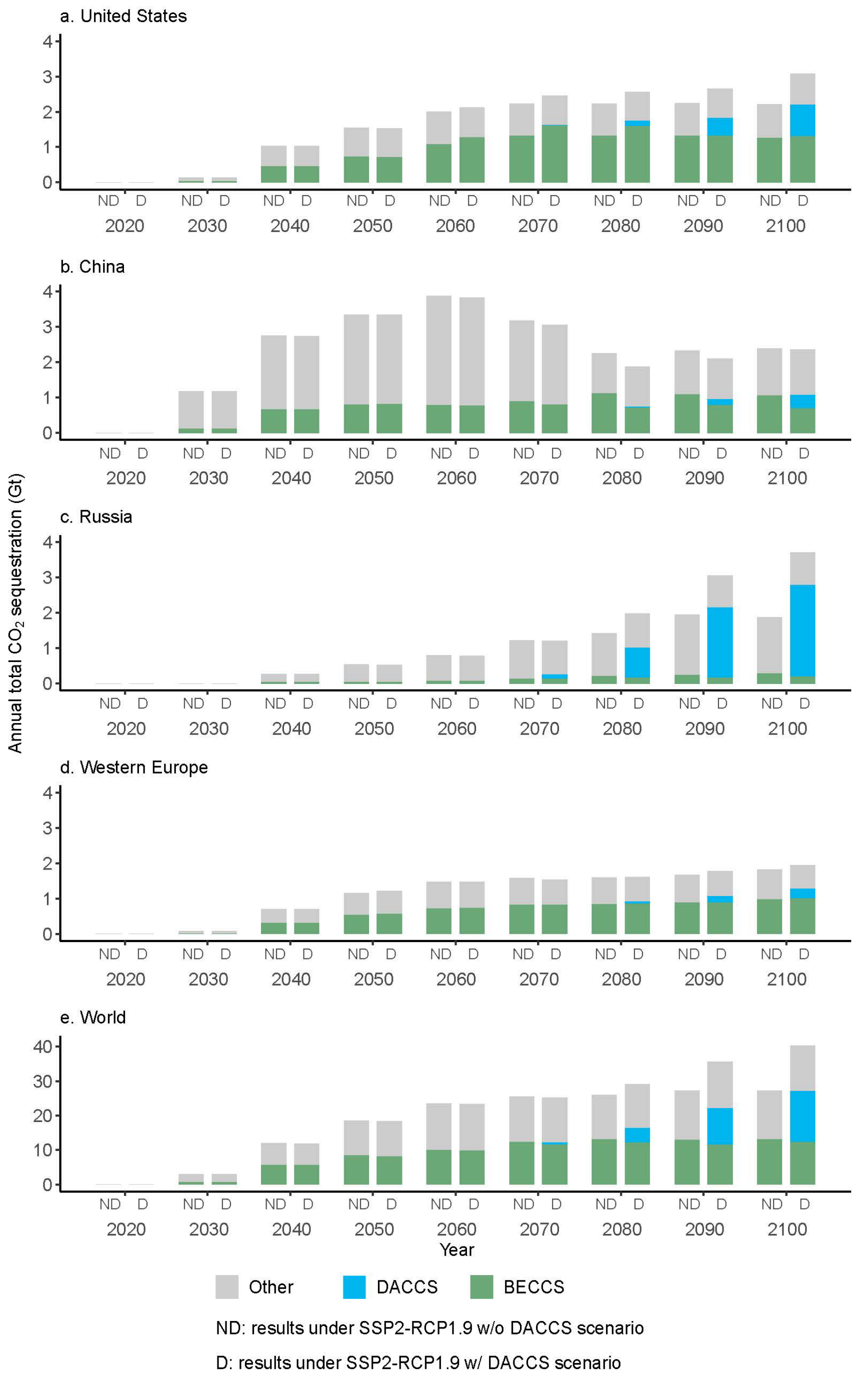

Supplementary Figure 8 | The annual total CO_2 sequestration in the United States (a), China (b), Russia (c), Western Europe (d) and the World as a whole (e) under SSP2-RCP1.9 w/o DACCS and SSP2-RCP1.9 w/ DACCS scenarios. There are two stacked bars for each year. The left bar ("ND") represents the results under SSP2-RCP1.9 w/o DACCS scenario. The right bar ("D") represents the results under SSP2-RCP1.9 w/ DACCS scenario. Different colors in the stacked bars represent different technologies used to sequester CO_2. BECCS is bioenergy with carbon capture and storage. DACCS is the direct air carbon capture and storage. Other includes carbon capture and storage (CCS) technologies applied in fossil fuel power plant, hydrogen, and industry sectors.

Supplementary Figure 9

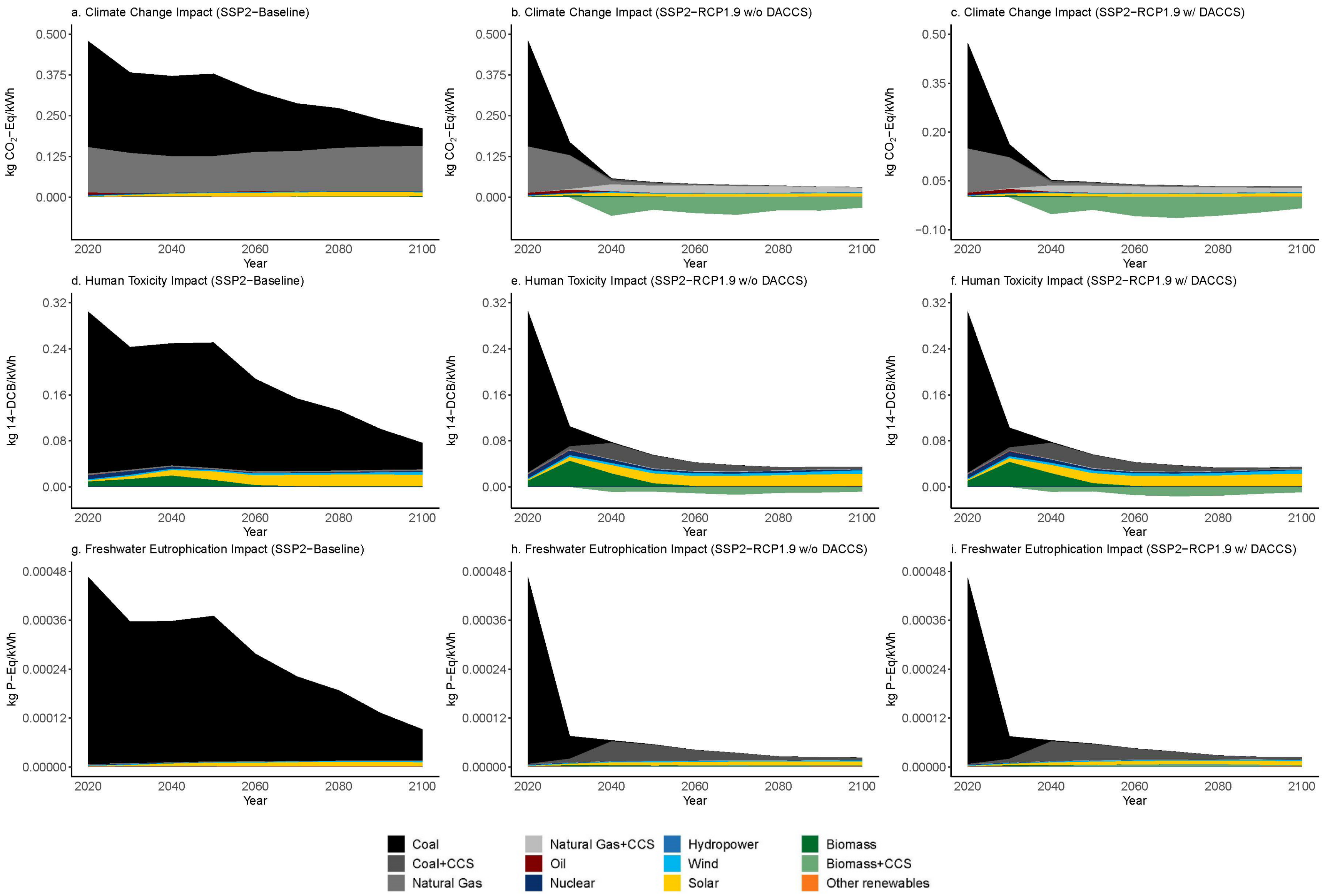

Figure to be continued

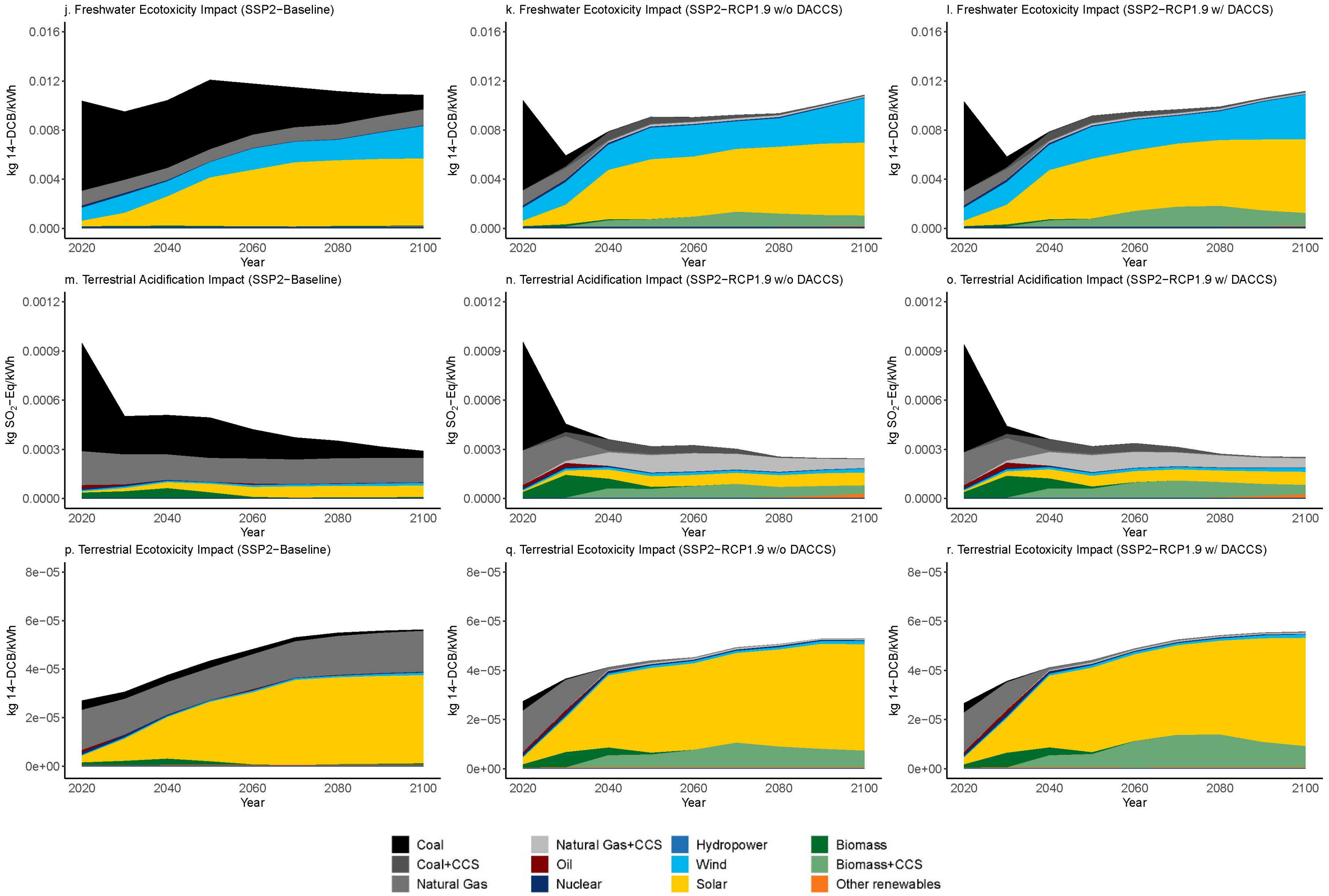

Figure to be continued

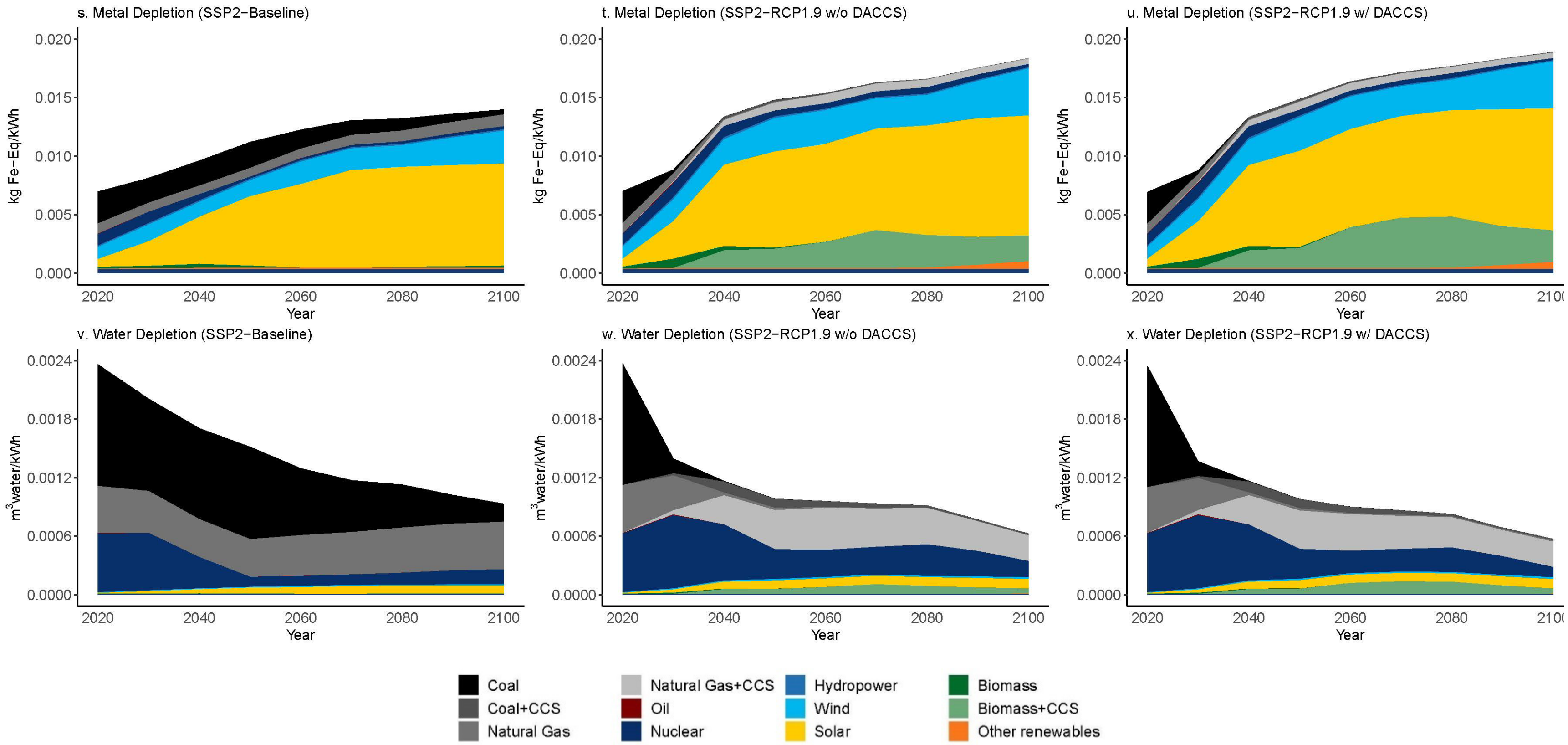

Supplementary Figure 9 | The life cycle environmental impact of 1 kWh US electricity generation from 2020 to 2100 under SSP2-Baseline, SSP2-RCP1.9 w/o DACCS and SSP2-RCP1.9 w/ DACCS scenarios. The stacked area represents the contribution of different electricity technologies to the total impacts. Impact categories include (a-c) climate change impact, (d-f) human toxicity impact, (g-i) freshwater eutrophication impact, (j-l) freshwater ecotoxicity impact, (m-o) terrestrial acidification impact, (p-r) terrestrial ecotoxicity impact, (s-u) metal depletion, (v-x) water depletion.

Supplementary Note 1: Life cycle inventory of two DACCS technologies

In this section, we provide the life cycle inventory (LCI) data of both solvent- and sorbent-based direct air capture (DAC) systems and subsequent compression and storage system. In the literature, some life cycle inventory (LCI) data of construction and operation of solvent-based DAC systems are missing, so we estimate some of the missing data based on engineering analysis of the material flow, equipment heuristics.

1. Material requirements and assumptions of solvent-based DAC

In this study, solvent-based DAC uses aqueous potassium hydroxide (KOH) solutions to capture atmospheric CO_2. The plant has an annual capacity of capturing 1 million metric tonnes (Mt) CO_2, with a lifetime of 20 years. The plant has four major components (contactor, pallet reactor, calciner, and slaker) and some auxiliary equipment. The material requirement data for constructing the air contactor of a solvent-based DAC plant are provided de Jong et al.[1], so these data are directly used as LCI of air contactor in this study. As for other components, the LCI data are missing in the literature, so their material requirements data were determined by sizing the equipment in accordance with the material flows in Keith et al (2018)[2] , engineering equipment heuristics, sizing approximations based on existing images and industry standards, and existing patents held by Carbon Engineering, which are described below.

Material requirements: Pellet reactor

The pellet reactor used by Carbon Engineering (CE) is a customized version of a wastewater treatment reactor designed by Royal HaskoningDHV, called the Crystalactor®[2]. From the renderings provided in Keith et al (2018), it was estimated that a 1 $MtCO_2$/year plant requires 48 pellet reactors. Each reactor shell is a stainless-steel cylinder with a height of 12 m and a diameter of 1.2 m. The actual shell may be more complex, but specific data could not be found. The wall thickness is assumed to be 0.022 m. This thickness comes from a safety factor of 3.5 given for a vertical vessel under pressure and with a diameter about 1 m, which account for potential corrosion risks[3]. The reactor shell has a 60-degree conical base with a diameter of 1.2 m. From this, the sidewall height of the base was calculated to be 3.6 m. The base thickness was assumed to be, again, 0.022 m due to the given conditions. The reactor lid was assumed to be a flat cap with a diameter of 1.2 m and a thickness of 0.022 m. Extra material required for a more complex lid was considered negligible. For the 48 vessel shells, bases, and lids, this results in a total stainless-steel requirement of 454 t/plant. Additional material required for automatic addition of seeds, washing and drying of seeds, and processing of fines were included in the Material requirements: Other equipment.

Material requirements: Calciner

The process proposed in Keith et al (2018). employs an oxy-fired fluidized bed calciner to produce calcium oxide (CaO) from calcium carbonate ($CaCO_{3)}$. The calcination step can be broken up into three steps: preheat, calcination and cooling:

The preheat step includes two cyclone heat exchangers (preheat 1 and preheat 2), that heat up solid stream (mostly $CaCO_3$) up to about 650°C. Since the material flow to the calciner is greater than 2,500 t/day, we assume that these cyclones are built as twin systems for a total of four cyclone preheaters. The diameters of the preheat 1 and preheat 2 are estimated at 8.1 m and 7.6 m, respectively (scaled based on the increased diameter and throughput presented in a previous study[4]). Further, the material

requirements were determined based on the circumference of the preheat cyclone and materials information from literature[5]. The preheat system also requires additional ducts leading from cyclone to cyclone, a draft fan for each set of cyclone preheaters, or a dip tube to increase material separation efficiency. These material requirements are included in Material requirements: Other equipment.

The calcination step in the Keith et al (2018) is based on fluidized bed calciner reactor, but we estimate the calciner material requirements using a rotary kiln, which is more widely deployed in industries such as cement making and pulp and paper production. While the two calciner configurations are different, the differences are assumed to have negligible effects on the material requirements for the calciner. Most of the material requirements for the calciner are from the metal body (in this case, steel) and the refractory (in this case, red brick refractory or alumina brick refractory). The material requirements for the calciner can be estimated using a cylindrical reactor with single refractory brick lining. The two primary material requirements are steel for the kiln shell and alumina bricks for the working refractory. Assuming the inside of the rotary kiln capable of processing 1,600 t raw material per day is 5.5 m, the working refractory layer is 0.254 m (or 10 inches) and the steel kiln shell is 0.04 m thick, this adds 1,000 t of steel and 2,050 t of red refractory brick for 1 Mt CO_2/year facility. These material requirements are then scaled linearly to achieve a throughput 3,960 t/day. Since the rotary kiln configuration is different than the fluidized bed, we then adjust the material requirements linearly using the projected cost. The cost of an oxy-fired rotary calciner is assumed to be \$120 million[6]., where the projected cost for the oxy-fired fluidized bed calciner is \$44 million from Keith et al (2018). Scaling these values yields material requirements of 910 t steel/plant and 1,856 t refractory/plant.

The cooling is a step after the calcination where the produced CaO is sent to an additional cyclone heat exchanger that preheats the incoming oxygen stream. Similar to the preheat, we assume that there are two, identical cyclones necessary on account of the high flow rate. The material requirements for the cyclone are estimated to be identical to cyclone preheat 2 with a radius of 7.6 m.

The complete material requirements for the calciner, broken down into the three sections, is shown in Supplementary Table 1.

Supplementary Table 1: Material requirements for the calciner unit of 1 Mt CO_2/year solvent-based DAC plant

Material Type	Material requirements (t/plant)			
	Preheat	Single-Lined Calciner	Cooling	Total
Steel	135	910	110	1155
Refractory Bricks	346	1856	168	2370
Concrete	195	0	95	290

Material Requirements: Steam Slaker

Two processes occur simultaneously inside of the slaker at 300°C: an exothermic reaction between CaO and water to produce $Ca(OH)_2$ and the heat transfer to solid $CaCO_3$ as a preheat to the calciner system[7]. The reaction vessel processes the solid CaO stream leaving the oxygen preheat (170 t/hr, 97% CaO, 3% K_2CO_3), water condensed from the steam turbine (70.2 t/hr), solids from the upstream $CaCO_3$ filter (306 t/hr, 98.2% $CaCO_3$, 1.8% K_2CO_3) and a recycle steam stream[2]. We assume this vessel is primarily steel (used to form the shell of the reactor) and refractory brick (used as insulation).

The solvent process presented in Keith et al[2] and detailed in Heidel and Rossi[7] uses a novel slaker configuration, mixing both the $CaCO_3$ streams and CaO streams to dry the $CaCO_3$ stream and recycle uncalcined material to the calciner, as well as create $Ca(OH)_2$. On account of the lower temperature requirement (300°C), we assume a refractory thickness of 6 inches (0.1524 m) total[8,9]. We assume that for the two calciners, there will be two slaking units, processing a total of 476 t/hr of solid material and roughly 70.2 t/hr of liquid/gaseous materials with equal distribution[2].

The outer shell of the slaker is assumed to be 40 mm, consistent with the metal shell thickness used for the calciner. The fluidization velocity is 1 m/s [2]. Assuming that the fluidizing medium is primarily steam, the specific volume of the superheated steam stream can be determined at 300°C and 1 bar (2.6 m^3/kg) to give a gas volumetric flowrate of 0.46 m^3/s. Therefore, we assume each slaker is a 0.4 m diameter cylindrical reaction vessel with an attached chamber for a recycle stream that is estimated using a factor 1.5 for the additional steel and refractory requirements based on the relative sizing of the cylindrical vessel to the recycle chamber. Assuming that the solid particle size entering the slacker experiences little to no particle size reduction occurring in the calciner, all particles will be roughly 0.85 mm in diameter when entering the slaker[2].

For most industrial fluidized beds, the length to diameter ratio lies between 3 and 16[10] and the typical reactor length is between 1 and 10 m[11]. For this analysis, we assume a bed length of 7.6 m (or a L/D ratio of 10). For the refractory thickness of 0.1524 m, the red silica brick requirement per vessel is roughly 9 t/reactor. For the steel thickness of 40 mm, the steel requirements are roughly 8 t/reactor. The total requirements for capturing 1 $MtCO_2$/year from air is shown in Supplementary Table 2.

Supplementary Table 2: Material requirements for the steam slaker of 1 Mt CO_2/year solvent-based DAC plant

Material type	Material requirements (t/plant)
Refractory Brick Required	18.0
Steel Required	15.8

The steam slaker requires additional equipment, such as a cyclone that separates the outlet gas stream, a baghouse unit, fines filter (separating $CaCO_3$ and $Ca(OH)_2$ post-slaker), heat exchangers and coolers. The material requirements for these smaller unit operations are included in Material requirements: Other equipment.

Material requirements: Other equipment

The material requirements for additional process equipment are assumed to be primarily concrete and steel. These additional units include the fines filter, quicklime mix tank, heat exchanger, and pumps, as well as any additional auxiliary equipment. To estimate the requirement for concrete, we assume 34% of the material costs from Keith et al. (2018) is distributed to concrete which is based on the American Institute of Steel Construction (AISC) construction material cost ratio to estimate the concrete requirements[12]. This is also similar to the methodology used in the Rhodium Group report Capturing New Jobs[13]. We assume a cost of concrete is \$61/t[14], consistent with the commodity price as of 2018. The resulting concrete requirements for the facility are calculated and outlined in Supplementary Table 3.

Supplementary Table 3: Concrete requirements of a 1 Mt CO_2/year solvent-based DAC plant

Module	Material cost[2] (Million $)	Total cost for cement (Million $)	Concrete requirements (t/plant)
Pellet Reactor	28.4	9.7	157,230
Calciner-Slaker	18.1	6.2	100,207
Others	31.8	10.8	176,054

The material requirements associated with piping and instrumentation are primarily steel and aluminum, and we estimate that separately based on a refinery configuration. The material requirements of any subsets (pipe, tubing, valves, fittings, and flanges) of the refinery (at three capacity levels: 10,000, 75,000, and 150,000 barrels/stream day) are provided in the Critical Materials Requirements for Petroleum Refining[15], then we calculated the steel requirements of the refinery with total capital cost of $6.06 billion (2018$) and a capacity of 50,000 barrel/stream day[16] using the scaling factor shown in Eq. 1 below.

$$Scale\ Factor = \frac{\log\left(\frac{Capacity\ B}{Capacity\ A}\right)}{\log\left(\frac{Material\ Requirements\ B}{Material\ Requirements\ A}\right)} \quad (1)$$

Then, we calculate the material requirements associated with piping and instrumentation of the solvent-based DAC as proportionate to that of the refinery plant (50,000 barrel/stream day) based on their capital costs (total capital cost are $6.06 billion for the refinery, total capital cost is $1.13 billion for solvent-based DAC[2], both on 2018$). The material requirements associated with piping and instrumentation are given in Supplementary Table 4.

Supplementary Table 4: Material requirements for piping and instrumentation of a 1 Mt CO_2/year solvent-based DAC plant

	Carbon Steel (t/plant)	Alloy Steel (t/plant)	Stainless Steel (t/plant)	Aluminum (t/plant)
Material requirements of piping and instrumentation	5,481	951	651	50

Chemical Requirements: Calcium Carbonate (CaCO3)

The initial requirements of $CaCO_3$ are required to start up the system. This is calculated using Figure 2 from Keith et al.[2]. The inlet $CaCO_3$ includes the three streams of $CaCO_3$ entering the pellet reactor: (1) $CaCO_3$ Seed (4.5 t/h), (2) $CaCO_3$ Makeup (3.4 t/h) and (3) $CaCO_3$ Seed from Calciner (6.0 t/h). The total startup $CaCO_3$ is 13.9 t/h for the duration of the startup period. The startup period discussed here is for the calcium loop (or calcining loop) and it primarily depends upon the calciner. Here, we assume a startup time of 24 hours to account for transit time through the calciner and associated equipment, which results in an initial $CaCO_3$ requirements to be 330 t. After the initial startup period, the annual make-up $CaCO_3$ is 3500 t/year[17]. Therefore, the annualized $CaCO_3$ consumption is 3,517 t (= 330t/20 + 3,500t).

Chemical Requirements: Potassium Hydroxide (KOH)

The initial KOH requirements are also directly dependent on the startup time of the system. Keith et al. [2] uses a 2 mol/L KOH solution that flows to both the contactor and post-combustion absorber at a flow rate of 35,000 t/hr. This is equivalent to roughly 3,000 t of KOH per hour for the duration of the startup period. We assume the same startup time of 24 hours to startup, including circulation from the contactors to the regeneration facility and the fluid residence time in the pellet reactors. In other words, the startup time accounts for the complete circulation of the fluid through the caustic recovery loop. So, the initial KOH requirement is 72,000 t. Although KOH is recycled through the system, but drift losses leads to an annual make-up KOH of 400 t/year[17]. Therefore, the annualized KOH consumption is 4,000 t (= 72,000t/20 + 400t).

Chemical Requirements: Water

The initial water requirements can also be estimated using the 35,000 t/h solvent flow to the contactor[2], which implies a water usage of roughly 31,000 t/h for the startup period of the contacting loop. With a startup time of 24 hours, the initial water usage is 744,000t. The temperature and relative humidity are used to estimate the water losses using the correlation given in Keith et al.[2]. We assumed a 60% relative humidity and 20°C, which resembles the temperature and humidity near Midland Texas November to March[18]. At these conditions, the evaporative losses are 3.8 t water/t CO_2 (430 t water/hour). By assuming a 90% operation capacity of DAC facility (7,884 hour/year), the annual make-up water is 3.4 Mt water/year. Therefore, the annualized water consumption is 3.44 Mt/year (= 0.74 Mt/20 + 3.4 Mt).

The overall material requirements for the construction of a 1 MtCO_2/year solvent-based DAC (except for air contactor) and its annualized chemical and water consumption of are summarized in Supplementary Table 5.

Supplementary Table 5: The overall material requirements for the construction of a 1 Mt CO_2/year solvent-based DAC plant (except for air contactor) and its annualized chemical and water consumption.

Material requirements for the construction (t/plant)						
Module	Concrete	Stainless-steel	Alloy steel*	Carbon steel	Aluminum	Refractory bricks
Pellet Reactors	157,230	454	0	0	0	0
Calciners-Slakers	100,497	0	1,171	0	0	2,388
Other Equipment	176,054	651	951	5,481	50	0
The annualized chemical and water consumption (t/plant·year)						
	KOH		CaCO$_3$		Water	
Chemical Requirements	4,000		3,517		3,440,000	

* Alloy steel combines both alloy steel and any other unspecified steel

The material and energy requirement data are converted to LCI data for solvent-based DAC systems and the subsequent compression and storage system based on 1 functional unit (capturing 1 t CO_2), which are summarized in Supplementary Table 6.

Supplementary Table 6. Life cycle inventory of the solvent-based DAC system (based on a sorbent-based DAC facility with annual capture capacity of 1 Mt CO_2 and lifetime of 20 years).

Input		Inventory Dataset[a]	Amount	Unit (per t CO_2 captured)
Construction				
Air contact	Concrete	RoW: market for concrete, normal	0.0067[b]	m^3
	Low-alloyed steel	GLO: market for steel, low-alloyed	0.27[b]	kg
	Stainless steel	RoW: steel production, chromium steel 18/8, hot rolled	0.0017[b]	kg
	Polyurethane	RoW: market for polyurethane, flexible foam	0.0005[b]	kg
	Glass fiber	GLO: market for glass fibre	0.0038[b]	kg
	Polypropylene	GLO: market for polypropylene, granulate	0.0008[b]	kg
	Polyvinyl Chloride	GLO: market for polyvinylchloride, bulk polymerised	0.76[b]	kg
Pellet reactor	Concrete	RoW: market for concrete, normal	0.0033[c]	m^3
	Stainless steel	RoW: steel production, chromium steel 18/8, hot rolled	0.023[c]	kg
Calciner slaker	Concrete	RoW: market for concrete, normal	0.0021[c]	m^3
	Low-alloyed steel	GLO: market for steel, low-alloyed	0.059[c]	kg
	Refractory brick	GLO: market for refractory, basic, packed	0.12[c]	kg
Other equipment	Concrete	RoW: market for concrete, normal	0.0037[c]	m^3
	Aluminium	GLO: market for aluminium, wrought alloy	0.0025[c]	kg
	Low-alloyed steel	GLO: market for steel, low-alloyed	0.048[c]	kg
	Stainless steel	RoW: steel production, chromium steel 18/8, hot rolled	0.033[c]	kg
	Carbon steel	GLO: market for steel, unalloyed	0.27[c]	kg
Operation				
Potassium hydroxide		GLO: market for potassium hydroxide	4.0[c]	kg
Calcium carbonate		RoW: market for limestone, crushed, for mill	3.5[c]	kg
Water		RoW: market for tap water	3,437[c]	kg
Electricity[d]		US: market group for electricity, medium voltage<br>CN: market group for electricity, medium voltage	345[b]	kWh

		ENTSO-E: market group for electricity, medium voltage<br>RU: market group for electricity, medium voltage<br>GLO: market group for electricity, medium voltage		
Heat[d]	Natural gas	RoW: heat production, natural gas, at industrial furnace >100kW	6,280[b]	MJ
	Biomethane	RoW: heat production, biomethane, at boiler condensing modulating <100kW[e]		
End-of-life[f]				
Concrete		RoW: treatment of waste concrete, inert material landfill	38[g]	kg
Steel		RoW: treatment of waste reinforcement steel, recycling	0.6	kg
Glass fiber		RoW: treatment of waste plastic, mixture, municipal incineration	0.0038	kg
Polyvinyl Chloride		RoW: treatment of waste polyvinylchloride, municipal incineration	0.76	kg
Polypropylene		RoW: treatment of waste polypropylene, municipal incineration	0.0008	kg
Polyurethane		RoW: treatment of waste polyurethane, municipal incineration	0.0005	kg
Refractory brick		RoW: treatment of waste brick, collection for final disposal	0.12	kg
Aluminium		RoW: treatment of aluminium scrap, post-consumer, prepared for recycling, at remelter	0.0023	kg
Potassium hydroxide		RoW: treatment of spent solvent mixture, hazardous waste incineration	4.0	kg
Calcium carbonate		RoW: treatment of limestone residue, inert material landfill	3.5	kg

Notes:

[a] The upstream and downstream inventory datasets are collected from ecoinvent 3.6.

[b] de Jong et al., 2019[1].

[c] Data collected based on the bottoms-up materials requirements analysis described in Supplementary Note 2.

[d] Here, we consider DAC system can be deployed in five regions, and the inventory data of electricity production are provided for these five regions: the United States (US), China (CN), Western Europe (ENTSO-E), Russia (RU) and World (GLO). Two heat supply options (natural gas and biomethane) are considered for solvent-based DAC system, and the inventory data of the heat supply are provided.

[e] ecoinvent 3.6 database does not include LCI data of the "RoW: heat production, biomethane, at boiler condensing modulating <100kW" process, but the LCI data of this process is included in the newest version (ecoinvent 3.7). Therefore, we collected the LCI datasets related this process from ecoinvent 3.7 and added them to ecoinvent 3.6 to create an extended ecoinvent 3.6. The data is also summarized in "4_LCI_biomethane_heat.xlsx" excel file in "LCI_data" folder.
[f] End-of-life (EoL) phase includes the treatment of materials used in construction and operation of DAC facility. We assume 85% steel (including low-alloyed and stainless steel, and steel pipe) used in the construction phase is recycled during end-of-life phase, and 90% aluminium used in the construction phase is recycled during end-of-life phase. All other materials (100%) are either incinerated or landfilled.
[g] Unit conversion of concrete from volume to mass by assuming the density of concrete as 2400 kg/m^3.

2. Life cycle inventory of sorbent-based DAC

For the sorbent system, the LCI data are collected from the work of Deutz and Bardow based on the Climeworks system[19]. We used the LCI data of the plant with an annual capacity of 100 kt CO_2/year and a lifetime of 20 years. The LCI data are summarized in Supplementary Table 7.

Supplementary Table 7. Life cycle inventory of the sorbent-based DAC system (based on a sorbent-based DAC module with annual capture capacity of 100 kt CO_2 and lifetime of 20 years).

Input		Inventory Dataset	Amount	Unit (per t CO_2 captured)
Construction				
Civil Engineering	Concrete for fundamentals	RoW: market for concrete, normal	0.004	m^3
	Steel for fundamentals	GLO: market for reinforcing steel	0.471	kg
Hall	Concrete for fundamentals	RoW: market for concrete, normal	0.003	m^3
	Steel for fundamentals	GLO: market for reinforcing steel	0.274	kg
	Steel structure	GLO: market for steel, low-alloyed	0.06	kg
	Insulation	GLO: market for stone wool	0.008	kg
Collector containers (without sorbent)	Carbon steel	GLO: market for steel, unalloyed	0.138	kg
	Stainless steel	RoW: steel production, chromium steel 18/8, hot rolled	0.112	kg
	Insulation	GLO: market for stone wool	0.005	kg
	Plastics (TPE)	RoW: market for polyurethane, rigid foam	0.006	kg
	Copper	GLO: market for copper	0.005	kg
	Aluminium	GLO: market for aluminium, wrought alloy	0.08	kg
	Paints, coating	RoW: market for alkyd paint, white, without solvent, in 60% solution state	0.005	kg
Process unit	Stainless steel	RoW: steel production, chromium steel 18/8, hot rolled	0.169	kg
	Low-alloyed steel	GLO: market for steel, low-alloyed	0.014	kg
	Insulation	GLO: market for polystyrene foam slab for perimeter insulation	0.047	kg
	Plastics (TPE)	RoW: market for polyurethane, rigid foam	0.005	kg
	Copper	GLO: market for copper	0.005	kg
Spare parts	Stainless steel	RoW: steel production, chromium steel 18/8, hot rolled	0.011	kg
	Low-alloyed steel	GLO: market for steel, low-alloyed	0.006	kg

Operation				
Amine-based sorbent (amine on silica)		Inventory data of amine-based sorbent is collected from literature and summarized in Supplementary Table 8	3.0	kg
Electricity		US: market group for electricity, medium voltage<br>CN: market group for electricity, medium voltage<br>ENTSO-E: market group for electricity, medium voltage<br>RU: market group for electricity, medium voltage<br>GLO: market group for electricity, medium voltage	500	kWh
Heat[a]	Heat pump	US: market group for electricity, medium voltage<br>CN: market group for electricity, medium voltage<br>ENTSO-E: market group for electricity, medium voltage<br>RU: market group for electricity, medium voltage<br>GLO: market group for electricity, medium voltage	5,400	MJ
	Biomethane	RoW: heat production, biomethane, at boiler condensing modulating <100kW		
End-of-life				
Concrete		RoW: treatment of waste concrete, inert material landfill	14.7	kg
Steel		RoW: treatment of waste reinforcement steel, recycling	1.07	kg
Aluminium		RoW: treatment of aluminium scrap, post-consumer, prepared for recycling, at remelter	0.072	kg
Plastics (TPE)		RoW: treatment of waste plastic, mixture, municipal incineration	0.058	kg
Copper		RoW: treatment of scrap copper, municipal incineration	0.01	kg
Stone wool		RoW: treatment of waste mineral wool, inert material landfill	0.013	kg

Amine-based sorbent (amine on silica)	RoW: treatment of spent anion exchange resin from potable water production, municipal incineration	3	kg

Notes:

[a] Two heat supply options (heat pump and biomethane) are considered for sorbent-based DAC system, and the inventory data of the heat supply are provided. The heat pump considered in this study has a coefficient of performance (COP) of 2.5, and it converts electricity into heat, so we use inventory of electricity production to represent the inventory of heat generation from heat pump. Heat requirement of sorbent-based DAC is 5,400 MJ/t CO_2 captured. If heat pump with COP of 2.5 is used to provide heat, the electricity consumption is 2,160 MJ/t CO_2 captured (600 kWh/t CO_2 captured).

3. Life cycle inventory of amine-based silica

The specific solid sorbent we choose for the sorbent-based DAC is amine-based silica, which can be synthesized by impregnate amines polyethylenimine (PEI) on solid silica gel. The LCI of amine-based silica is collected from literature (taking average between the best- and worst-case)[19] and summarized in Supplementary Table 8 based on the composition that 1 kg amine-based silica requires of 0.64kg silica gel and 0.36 kg PEI[20] (The data is also summarized in "3_LCI_amine_based_sorbent.xlsx" excel file in "LCI_data" folder).

Supplementary Table 8. Life cycle inventory of 1 kg amine-based silica.

Input	Dataset	Amount	Unit (per 1 kg amine-based silica)
Silica gel (64% in mass composition of 1 kg amine-based silica)[a]			
Sodium silicate	RoW: market for sodium silicate, solid	0.13	kg
Sulfuric acid	RoW: market for sulfuric acid	0.02	kg
Thermal energy	RoW: market for heat, central or small-scale, natural gas	0.63	MJ
Water	RoW: market for water, deionised	1.29	kg
Wastewater treatment	RoW: treatment of wastewater, average, capacity 1E9l/year	1.12	m^3
Particulates (<2.5 um)	Emission to air	0.000042	kg
PEI (36% in mass composition of 1 kg amine-based silica)			
Ethanolamine	GLO: market for monoethanolamine	0.71	kg
Sulfuric acid	RoW: market for sulfuric acid	1.14	kg
Sodium hydroxide	GLO: market for sodium hydroxide, without water, in 50% solution state	1.00	kg
Hydrochloric acid	RoW: market for hydrochloric acid, without water, in 30% solution state	0.07	kg
Ethanol	GLO: market for ethanol, without water, in 99.7% solution state, from fermentation	1.24	kg
Diethyl ether	RoW: market for diethyl ether, without water, in 99.95% solution state	14.84	kg
Water	RoW: market for water, deionised	5.24	kg
Electricity	GLO: market group for electricity, low voltage	0.12	kWh
Thermal energy	RoW: market for heat, central or small-scale, natural gas	2.69	MJ
Sodium sulfate (co-product as output)	RoW: market for sodium sulfate, anhydrite	1.65	kg
Unreacted raw materials and solvents	RoW: treatment of spent solvent mixture, hazardous waste incineration	0.54	kg
End-of-Life			

amine-based silica	RoW: treatment of spent anion exchange resin from potable water production, municipal incineration	0.36[b]	kg

Notes:

[a] Silica gel is assumed to be recycled with a rate of 95 %[19], so the material and energy flows in this table (for silica gel) have factored in the recycling rate, meaning the amounts are 5% of the original required amount.

[b] This process only applies for the PEI, because 95% of silica are recycled.

4. Life cycle inventory of pipeline transport and storage system

Once the captured CO_2 is release from the DAC system, we assume the CO_2 flow will be compressed through a compressor to 11 MPa and then transported through a pipeline to the storage site. The length of the transport pipeline is assumed to be 50 km. At the storage site, the CO_2 will be further compressed to 15 MPa and injected into a geological reservoir through wells with the depth of 3 km each. The LCI data of transport and storage system are collected from a previous study[21] and summarized in Supplementary Table 9.

Supplementary Table 9. Life cycle inventory of compression, pipeline transport and storage system.

Input		Dataset	Amount	Unit (per t CO_2 compressed and stored)
		Construction		
Compression facility	Concrete	RoW: market for concrete, normal	0.000001	m^3
	Copper	GLO: market for copper	0.0001	kg
	Low alloyed steel	GLO: market for steel, low-alloyed	0.001	kg
	Polyethylene	GLO: market for polyethylene, low density, granulate	0.0003	Kg
	Diesel	RoW: market for diesel	0.032	MJ
	Electricity	US: market group for electricity, medium voltage<br>CN: market group for electricity, medium voltage<br>ENTSO-E: market group for electricity, medium voltage<br>RU: market group for electricity, medium voltage<br>GLO: market group for electricity, medium voltage	0.001	kWh
Pipeline transport	Sand	RoW: market for sand	1.04	m^3
	Reinforcing steel	GLO: market for reinforcing steel	0.13	kg
	Steel pipes	GLO: market for drawing of pipe, steel	0.13	kg
	Bitumen	GLO: market for bitumen seal	0.0012	Kg
	Polyethylene	GLO: market for polyethylene, low density, granulate	0.0025	kg
	Diesel	RoW: market for diesel	1.8	MJ
	Transport	RoW: market for transport, freight, lorry, unspecified	0.12	t*km
Geological storage	Well construction	GLO: market for onshore well, oil/gas	8.2E-08	km
	Sand	RoW: market for sand	3.3	kg

Un-alloyed steel	GLO: market for steel, unalloyed	0.017	kg
Low alloyed steel	GLO: market for steel, low-alloyed	0.037	kg
Concrete	RoW: market for concrete, normal	0.000048	m^3
Copper	GLO: market for copper	0.0019	kg
Transport	RoW: market for transport, freight, lorry, unspecified	0.34	t*km
Operation			
Electricity	US: market group for electricity, medium voltage<br>CN: market group for electricity, medium voltage<br>ENTSO-E: market group for electricity, medium voltage<br>RU: market group for electricity, medium voltage<br>GLO: market group for electricity, medium voltage	118	kWh
End-of-life			
Concrete and sand	RoW: treatment of waste concrete, inert material landfill	4.4	kg
Steel	RoW: treatment of waste reinforcement steel, recycling	0.26	kg
Copper	RoW: treatment of scrap copper, municipal incineration	0.002	kg
Polyethylene	RoW: treatment of waste polyethylene, municipal incineration	0.0028	kg
Bitumen	RoW: treatment of waste bitumen, sanitary landfill	0.0012	kg

Supplementary Note 2: Technology learning assumption of solvent- and sorbent-based DAC

Solvent-based DAC approach uses a liquid solvent and high surface area packing material to capture ambient CO_2. Current applications require strong bases, such as NaOH and KOH, with uptake of 3.1E-5 mol $CO_2/cm^2\cdot$second (0.52 mol CO_2/minute$\cdot m^3$)[22]. If innovative approaches can increase the uptake rate to 7.0E-5 mol $CO_2/cm^2\cdot$second (1.18 mol CO_2/minute$\cdot m^3$), this would result in a 2.3 times increase in the uptake rate. This could result from an improved packing material that increases the solvent's exposed surface area, or by the development of novel liquid solvents with higher uptake capacities. The increase in uptake translates to a roughly proportional decrease in the bed depth of the contactor and a roughly 56% decrease in the cost of the contactor unit. The decreased bed depth additionally causes a reduction in the system fan power by the same percentage. Then, we also assumed that increased deployment improves the system thermal efficiency, which results in a reduction of system thermal energy demand by 2.4 GJ/tCO_2 for a total energy requirement of 6 GJ/tCO_2[23]. As described in the 2019 National Academies of Sciences Engineering and Medicine (NASEM) report on negative emissions technologies, the inlet surface area dimensions of contactor are assumed to be 20 m by 200 m in both uptake scenarios[24]. Instead, the bed depth is varied. The cost reduction for the contactor is proportional to the size change of contact unit.

For initial cost of solvent-based DAC, we used the upper bound cost data from the NASEM report, which is \$264/tCO_2 (capital cost = \$151/t CO_2, operating cost = \$113/t CO_2, with a capacity of 1 Mt CO_2/year)[24]. To estimate the how these costs will come down, we applied the 56% decrease to the capital cost of contactor, and we assumed that innovation in other unit operations will result in achievement of the lower bound capital costs as described in the NASEM report. So, we developed the theoretical minimum capital cost at \$67/t CO_2 (44% of today's capital cost). (A capital recovery factor of 12.4% was used annualize the capital costs of the system). Then, we adopted a learning rate range (1% to 15%)[25] from various existing emerging technologies to project the reduction of capital cost. Under 10% learning rate, the capital cost approximates to the minimum \$67/t CO_2 when the learning effect is saturated, so the 10% is chosen as the reference learning rate for capital costs of solvent-based DAC. Furthermore, we adopted a range of learning rate (5%–15%) for the capital cost of solvent-based DAC from the literature to reflect uncertainty in the actual learning rate[26]. Similarly, to adjust the operating cost, the fan energy was reduced by 56% and a reduction of 2.4 GJ/tCO_2 is applied to the thermal energy demand, which give the minimum operating costs at \$56/tCO_2 (50% of today's operating cost). As for the learning rate, a few previous studies adopted a conservative assumption by considering a fixed operating cost (no learning) over time[25,26], so here we assumed an reference learning rate of 2.5% for the operating cost, with a range varying from 0% (no learning) to 5%.

Sorbent-based DAC uses solid sorbents to uptake CO_2 in a batch-wise process. The first area for innovation lies within the sorbent itself. Sorbents designed with higher uptake rate and longer lifetimes can reduce the amount of sorbent necessary in the DAC contactors. Since the sorbent makes up roughly 80% of the system's capital cost[27], this has a huge impact on the process economics. The current uptake observed in commercial sorbents is 2.5 mol CO_2/kg over 3,000 s (3.53 mol CO_2/minute$\cdot m^3$)[22,24]. Higher capacity sorbents are described to reach an uptake of 3.4 mol CO_2/kg over 12 hours in an aminopolymer-impregnated silica sorbent [28]. If future innovation can lead to similar uptakes in 3,000 s, this increases the specific uptake to 4.76 mol CO_2/minute$\cdot m^3$. Additionally, we assume that the average lifetime will lengthen from 0.5 years to 2 years [24], which reduces the amount of makeup sorbent by four times the original value. The joint impact of increased uptake and longer sorbent lifetime results in a

sorbent cost decrease of roughly 82%, resulting in a 74% decrease in the overall capital costs compared to the middle case NASEM report. The cost per unit sorbent is assumed to remain consistent at $50/kg. Due to the potential reduction of sorbent use described above, we assume the learning rate and the theoretical minimum value of sorbent use will be the same as those applied for capital cost of sorbent DACCS.

The cost data from scenario 4 – High in the same NASEM represent the cost of sorbent-based DAC with the plant capacity 1 Mt CO_2/year too. After adjusting the capital cost to represent an economic lifetime of 10 years and a 11.6% discount rate, the total cost is $386/t$CO_2$ (capital cost = $364/t CO_2, operating cost = $22/t CO_2). In this study, life cycle inventory data we used for sorbent-DAC is based on plant capacity of 0.1 Mt CO2/year, so we further estimated the initial cost of a sorbent-DAC with the capacity of 0.1 Mt CO2/year using the learning curve approach. We assumed the plant with the capacity of 4,000t CO_2/year to be $900/t CO_2 (by averaging the costs of 4,000t CO_2/year sorbent-based plant from multiple sources[25,29]), and then the cost of a plant with the capacity of 1 Mt CO2/year was assumed to be $386/t$CO_2$. We fitted these data into a regression of one factor learning curve equation, and then we estimated the cost of a sorbent-DAC with the capacity of 0.1 Mt CO_2/year to be $550/t$CO_2$ (capital cost = $518/t CO_2, operating cost = $32/t CO_2), and we use this cost as the initial cost of sorbent-based DAC plant (with the capacity of 0.1 Mt CO_2/year).

The aforementioned changes to the sorbent capacity and lifetime coupled to the assumption that other process innovations will shift the capital cost from scenario 4 – High to scenario-2 Low described in the NASEM report result in a reduction of the system's levelized capital costs by 82% ($101/t$CO_2$). For the operating cost, we also shift it from scenario 4 – High to scenario 2 – Low, resulting in a minimum operating cost of $16/t$CO_2$ (50% of initial operating cost). Using similar method for developing the learning rate as described in the solvent-based DAC, we adopted a learning rate range for sorbent-based DAC, which is from 5% to 20%. The higher range is chosen is because, compared to solvent-based DAC which is highly integrated and large-scale, sorbent-based DAC relies on standardized and modular units, which can be mass-produced and deployed, and therefore enables fast iteration and learning[26]. The learning rate chosen to best represent the capital costs of sorbent-based DAC is 15% (as the reference learning rate), and the uncertainty range was set to be 10%–20%[26]. For the operating cost, we used the same learning rate as the solvent-based DAC, with the reference rate being 2.5% and variation range being 0%–5%.

Here, we also assume that the subsequent CO_2 transport and storage facilities will follow the same learning rates as the corresponding solvent- and sorbent-based DAC systems. The selected learning rates and theoretical minimum costs of both solvent- and sorbent-based DACCS are summarized in the Supplementary Table 10. Because the effects of technology learning on material and energy use of DACCS are so far missing in the published literature, we assume the changes of material and energy consumption are proportional to the changes of the costs of DACCS technologies. Therefore, we used these learning rates and their theoretical minimum values to estimate the corresponding material and energy uses that are related to these cost metrics.

Supplementary Table 10. Assumed reference learning rate (and their uncertainty ranges), theoretical minimum value of capital and operational costs (also representing the material and energy consumption associated to these cost metrics) of DACCS technologies.

Technology type		Solvent-based DACCS	Sorbent-based DACCS
Learning rate[a]	Capital cost	10% (5%–15%)	15% (10%–20%)
	Operational cost	2.5% (0%–5%)	2.5% (0%–5%)
Theoretical minimum values (percentage of initial cost)	Capital cost	44%	18%
	Operational cost	50%	50%

Note:
[a] Numbers in the parenthesis represent the uncertainty ranges

There is no concrete way to imply the learning rate between two points. This approximation has DACCS approaching a theoretical minimum cost at different rates. Future innovation is unpredictable and, therefore, the actual minimum cost may be different from the estimated values.

Supplementary Table 11. Cumulative DACCS deployment and material and energy use factors of DACCS technologies from 2020 to 2100

Year	Cumulative DACCS deployment (Gt/yr)[a]	Solvent-based DACCS						Sorbent-based DACCS					
		Material and energy use factor (capital cost)[b]			Material and energy use factor (operational cost)[b]			Material and energy use factor (capital cost)[b]			Material and energy use factor (operational cost)[b]		
		Slow	Reference	Fast	Slow	Reference	Fast	Slow	Reference	Fast	Slow	Reference	Fast
2020	0	1.00	1.00	1.00	1.00	1.00	1.00	1.00	1.00	1.00	1.00	1.00	1.00
2025	0	1.00	1.00	1.00	1.00	1.00	1.00	1.00	1.00	1.00	1.00	1.00	1.00
2030	0	1.00	1.00	1.00	1.00	1.00	1.00	1.00	1.00	1.00	1.00	1.00	1.00
2035	0	1.00	1.00	1.00	1.00	1.00	1.00	1.00	1.00	1.00	1.00	1.00	1.00
2040	0	1.00	1.00	1.00	1.00	1.00	1.00	1.00	1.00	1.00	1.00	1.00	1.00
2045	0	1.00	1.00	1.00	1.00	1.00	1.00	1.00	1.00	1.00	1.00	1.00	1.00
2050	0.003	1.00	1.00	1.00	1.00	1.00	1.00	1.00	1.00	1.00	1.00	1.00	1.00
2055	0.021	0.92	0.86	0.80	1.00	0.97	0.93	0.79	0.70	0.62	1.00	0.97	0.93
2060	0.069	0.88	0.79	0.71	1.00	0.95	0.89	0.69	0.57	0.48	1.00	0.95	0.90
2065	0.209	0.85	0.73	0.65	1.00	0.93	0.86	0.61	0.48	0.39	1.00	0.93	0.86
2070	0.680	0.82	0.69	0.60	1.00	0.91	0.83	0.54	0.41	0.32	1.00	0.91	0.83
2075	1.758	0.79	0.65	0.57	1.00	0.90	0.81	0.49	0.36	0.28	1.00	0.90	0.81
2080	3.284	0.77	0.63	0.55	1.00	0.89	0.80	0.46	0.34	0.27	1.00	0.89	0.80
2085	5.036	0.76	0.62	0.54	1.00	0.88	0.79	0.44	0.32	0.25	1.00	0.88	0.79
2090	6.906	0.76	0.61	0.53	1.00	0.88	0.78	0.43	0.31	0.25	1.00	0.88	0.78
2095	8.828	0.75	0.61	0.53	1.00	0.87	0.77	0.42	0.31	0.24	1.00	0.87	0.78
2100	10.671	0.75	0.60	0.53	1.00	0.87	0.77	0.42	0.30	0.24	1.00	0.87	0.77

Note:

[a] The cumulative DACCS deployment are calculated by dividing the global cumulative DACCS deployment results (IMAGE output under SSP2-RCP1.9 w/ DACCS scenario) by half, because we assume solvent- and sorbent-based DACCS contribute the same to the DACCS deployment globally.

[b] The material and energy use factors are developed based on cumulative DACCS deployment, learning rates, and theoretical minimum value of capital and operational costs (Supplementary Table 10). The material and energy use factors are 1 in the starting year (2020), and then factors of the following year are expressed as the ratios relative to those in 2020 as the technology learning starts. By multiplying these material and energy use factors to the actual amount of material and energy uses of DACCS systems in 2020, we can get the dynamic material and energy use data of DACCS, which can be used as LCI data to evaluate the prospective environmental impacts of DACCS with the consideration of technology learning. The results under the columns named by "Reference" were estimated based on the reference learning rates in Supplementary Table 10. The results under columns named by "Slow" and "Fast" were estimated based on the lower bound (slow) and upper bound (fast) learning rates, respectively, and that is why the results under "Slow" column (representing slow learning) have higher numeric values, while the results under "Fast" column (representing fast learning) have lower numeric values.

Supplementary Note 3: Technologies map between IMAGE 3.2 and ecoinvent v3.6

Given the differences of generation technologies between IMAGE and ecoinvent database, here we adopted the matching list from a previous study[30] to map the available technologies in both data sources (Supplementary Table 12). Most of the generation technologies in IMAGE can be linked to one or more processes in the ecoinvent 3.6, which provides their LCI data. But there are some electricity generation technologies that appears in IMAGE scenarios but are missing in ecoinvent databases, so we imported their LCI data from external data sources to extend our the ecoinvent database (indicated as foot notes in Supplementary Table 12).

Supplementary Table 12. Technologies map between IMAGE 3.2 and ecoinvent v3.6

IMAGE technology	Ecoinvent processes
Solar PV power (central)	electricity production, photovoltaic, 570kWp open ground installation, multi-Si
Solar PV power (decentral/residential)	electricity production, photovoltaic, 3kWp facade installation, multi-Si, laminated, integrated,<br>electricity production, photovoltaic, 3kWp facade installation, multi-Si, panel, mounted,<br>electricity production, photovoltaic, 3kWp facade installation, single-Si, laminated, integrated,<br>electricity production, photovoltaic, 3kWp facade installation, single-Si, panel, mounted,<br>electricity production, photovoltaic, 3kWp flat-roof installation, multi-Si,<br>electricity production, photovoltaic, 3kWp flat-roof installation, single-Si,<br>electricity production, photovoltaic, 3kWp slanted-roof installation, a-Si, laminated, integrated,<br>electricity production, photovoltaic, 3kWp slanted-roof installation, a-Si, panel, mounted,<br>electricity production, photovoltaic, 3kWp slanted-roof installation, CdTe, laminated, integrated,<br>electricity production, photovoltaic, 3kWp slanted-roof installation, CIS, panel, mounted,<br>electricity production, photovoltaic, 3kWp slanted-roof installation, multi-Si, laminated, integrated,<br>electricity production, photovoltaic, 3kWp slanted-roof installation, multi-Si, panel, mounted,<br>electricity production, photovoltaic, 3kWp slanted-roof installation, ribbon-Si, laminated, integrated,<br>electricity production, photovoltaic, 3kWp slanted-roof installation, ribbon-Si, panel, mounted,<br>electricity production, photovoltaic, 3kWp slanted-roof installation, single-Si, laminated, integrated
Concentrated solar power	electricity production, solar thermal parabolic trough, 50 MW,<br>electricity production, solar tower power plant, 20 MW

Onshore wind power	electricity production, wind, <1MW turbine, onshore,<br>electricity production, wind, 1-3MW turbine, onshore,<br>electricity production, wind, >3MW turbine, onshore
Offshore wind power	electricity production, wind, 1-3MW turbine, offshore
Wave power[a]	electricity production, wave
Hydro power	electricity production, hydro, reservoir, alpine region,<br>electricity production, hydro, reservoir, non-alpine region,<br>electricity production, hydro, reservoir, tropical region,<br>electricity production, hydro, run-of-river
Other renewables (tidal and geothermal power)	electricity production, deep geothermal
Nuclear	electricity production, nuclear, boiling water reactor,<br>electricity production, nuclear, pressure water reactor, heavy water moderated,<br>electricity production, nuclear, pressure water reactor
Coal steam turbine	electricity production, hard coal,<br>electricity production, lignite,<br>electricity production, peat,<br>electricity production, hard coal, conventional,<br>electricity production, hard coal, supercritical
Oil steam turbine	electricity production, oil
Natural gas open cycle turbine	electricity production, natural gas, conventional power plant
Biomass steam turbine	electricity production, wood, future
Integrated gasification combined cycle[b]	Electricity, at power plant/hard coal, IGCC, no CCS/2025,<br>Electricity, at power plant/lignite, IGCC, no CCS/2025
Oil combined cycle	electricity production, oil<br>(Use copy of Oil steam turbine here as Oil combined cycle does not exist in ecoinvent)
Natural gas combined cycle	electricity production, natural gas, combined cycle power plant
Biomass combined cycle[b]	Electricity, at BIGCC power plant 450MW, no CCS/2025
Coal with CCS[b]	Electricity, at power plant/hard coal, pre, pipeline 200km, storage 1000m/2025,<br>Electricity, at power plant/lignite, pre, pipeline 200km, storage 1000m/2025,<br>Electricity, at power plant/hard coal, post, pipeline 200km, storage 1000m/2025,<br>Electricity, at power plant/lignite, post, pipeline 200km, storage 1000m/2025,<br>Electricity, at power plant/lignite, oxy, pipeline 200km, storage 1000m/2025,<br>Electricity, at power plant/hard coal, oxy, pipeline 200km, storage 1000m/2025
Oil with CCS	Electricity, at power plant/hard coal, pre, pipeline 200km, storage 1000m/2025,<br>Electricity, at power plant/lignite, pre, pipeline 200km, storage

	1000m/2025,<br>Electricity, at power plant/hard coal, post, pipeline 200km, storage 1000m/2025,<br>Electricity, at power plant/lignite, post, pipeline 200km, storage 1000m/2025,<br>Electricity, at power plant/lignite, oxy, pipeline 200km, storage 1000m/2025,<br>Electricity, at power plant/hard coal, oxy, pipeline 200km, storage 1000m/2025,<br>Electricity, at power plant/natural gas, pre, pipeline 200km, storage 1000m/2025,<br>Electricity, at power plant/natural gas, post, pipeline 200km, storage 1000m/2025<br>(the LCI data of oil with CCS is not available, so we just use the dataset of coal and natural gas with CCS as a proxy)
Nature gas with CCS[b]	Electricity, at power plant/natural gas, pre, pipeline 200km, storage 1000m/2025,<br>Electricity, at power plant/natural gas, post, pipeline 200km, storage 1000m/2025
Biomass with CCS[b]	Electricity, at BIGCC power plant 450MW, pre, pipeline 200km, storage 1000m/2025
Coal combined heat and power (CHP)	heat and power co-generation, hard coal,<br>heat and power co-generation, lignite
Oil CHP	heat and power co-generation, oil
Nature gas CHP	heat and power co-generation, natural gas, combined cycle power plant, 400MW electrical,<br>heat and power co-generation, natural gas, conventional power plant, 100MW electrical,<br>heat and power co-generation, natural gas, 500kW electrical, lean burn
Biomass CHP	heat and power co-generation, wood chips, 6667 kW, state-of-the-art 2014,<br>heat and power co-generation, wood chips, 6667 kW
Coal CHP with CCS	Electricity, at power plant/hard coal, pre, pipeline 200km, storage 1000m/2025,<br>Electricity, at power plant/lignite, pre, pipeline 200km, storage 1000m/2025,<br>Electricity, at power plant/hard coal, post, pipeline 200km, storage 1000m/2025,<br>Electricity, at power plant/lignite, post, pipeline 200km, storage 1000m/2025,<br>Electricity, at power plant/lignite, oxy, pipeline 200km, storage 1000m/2025,<br>Electricity, at power plant/hard coal, oxy, pipeline 200km, storage 1000m/2025<br>(the LCI data of coal CHP with CCS is not available, so we just use the dataset of coal with CCS as a proxy)

Oil CHP with CCS	Electricity, at power plant/hard coal, pre, pipeline 200km, storage 1000m/2025,<br>Electricity, at power plant/lignite, pre, pipeline 200km, storage 1000m/2025,<br>Electricity, at power plant/hard coal, post, pipeline 200km, storage 1000m/2025,<br>Electricity, at power plant/lignite, post, pipeline 200km, storage 1000m/2025,<br>Electricity, at power plant/lignite, oxy, pipeline 200km, storage 1000m/2025,<br>Electricity, at power plant/hard coal, oxy, pipeline 200km, storage 1000m/2025,<br>Electricity, at power plant/natural gas, pre, pipeline 200km, storage 1000m/2025,<br>Electricity, at power plant/natural gas, post, pipeline 200km, storage 1000m/2025<br>(the LCI data of oil CHP with CCS is not available, so we just use the dataset of coal and natural gas with CCS as a proxy as a proxy)
Natural gas CHP with CCS	Electricity, at power plant/natural gas, pre, pipeline 200km, storage 1000m/2025,<br>Electricity, at power plant/natural gas, post, pipeline 200km, storage 1000m/2025<br>(the LCI data of natural gas CHP with CCS is not available, so we just use the dataset of coal and natural gas with CCS as a proxy as a proxy)
Biomass CHP with CCS[b]	Electricity, at wood burning power plant 20 MW, truck 25km, post, pipeline 200km, storage 1000m/2025

Note:

[a] LCI of wave electricity generation is collected based on an attenuator-type floating oscillating body system wave energy converter with a capacity of 750kW[31]. The LCI data is also summarized in "1_LCI_wave_electricity.xlsx" excel file in "LCI_data" folder.

[b] We adopted the LCI of fossil fuel with CCS that is summarized in a previous study[30]. The LCI data is also summarized in "2_LCI_CCS.xlsx" excel file in "LCI_data" folder.

References

1. de Jonge, M. M. J., Daemen, J., Loriaux, J. M., Steinmann, Z. J. N. & Huijbregts, M. A. J. Life cycle carbon efficiency of Direct Air Capture systems with strong hydroxide sorbents. *International Journal of Greenhouse Gas Control* **80**, 25–31 (2019).
2. Keith, D. W., Holmes, G., St. Angelo, D. & Heidel, K. A Process for Capturing CO2 from the Atmosphere. *Joule* **2**, 1573–1594 (2018).
3. Burr, A. & Cheatham, J. *Mechanical Design and Analysis*. (1995).
4. European Cement Research Academy (ECRA). ECRA CCS Project: Report on Phase IV.A (Technical Report). (2016).
5. Sharrcem - Titan Antea Cement. *Scaffolding inside the Pre-heater Cyclones & Calciner*. (2012).
6. American Physical Society (APS). *Direct Air Capture of CO_2 with Chemicals*. (2011).
7. Heidel, K. R. & Rossi, R. A. High Temperature Hydrator. (2017).
8. FEECO. Direct-Fired Rotary Kilns. https://feeco.com/rotary-kilns/ (2016).
9. Ebbenis, A. & Carlson, C. Everything You Need to Know on Rotary Kiln Refractory. https://feeco.com/everything-you-need-to-know-on-rotary-kiln-refractory/ (2020).
10. Shaul, S., Rabinovich, E. & Kalman, H. Generalized flow regime diagram of fluidized beds based on the height to bed diameter ratio. *Powder Technology* **228**, 264–271 (2012).
11. Cocco, R., Reddy, S. B. & Knowlton, K. T. *Back to Basics Introduction to Fluidization*. (2014).
12. American Institute of Steel Construction (AISC). *Structural Steel: An Industry Overview*. (2018).
13. Larsen, J., Herndon, W., Analyst, S. & Hiltbrand, G. *Capturing New Jobs: The employment opportunities associated with scale-up of Direct Air Capture (DAC) technology in the US*. (2020).
14. Home Advisor. 2020 Concrete Delivery Costs. https://www.homeadvisor.com/cost/outdoor-living/deliver-concrete/.
15. Wells, E. *Criticial materials requirements for petroleum refining*. (1966).

16. Canadian Fuels Association. *The Economics of Petroleum Refining into fuels and other value added products*. (2013).
17. National Academies of Sciences, E. *Negative emissions technologies and reliable sequestration: a research agenda.* (National Academies Press, 2018).
18. Weather Spark. Average Weather in Midland, Texas, United States, Year Round. https://weatherspark.com/y/4333/Average-Weather-in-Midland-Texas-United-States-Year-Round (2017).
19. Deutz, S. & Bardow, A. Life-cycle assessment of an industrial direct air capture process based on temperature–vacuum swing adsorption. *Nature Energy* **6**, 203–213 (2021).
20. Wurzbacher, J. A., Gebald, C. & Steinfeld, A. Separation of CO2 from air by temperature-vacuum swing adsorption using diamine-functionalized silica gel. *Energy Environ. Sci.* **4**, 3584 (2011).
21. Koornneef, J., van Keulen, T., Faaij, A. & Turkenburg, W. Life cycle assessment of a pulverized coal power plant with post-combustion capture, transport and storage of CO2. *International Journal of Greenhouse Gas Control* **2**, 448–467 (2008).
22. Wilcox, J. *Carbon Capture*. (Springer, 2012).
23. Zeman, F. Reducing the cost of ca-based direct air capture of CO_2. *Environmental Science and Technology* **48**, 11730–11735 (2014).
24. National Academies of Sciences Engineering and Medicine (NASEM). *Negative Emissions Technologies and Reliable Sequestration: A Research Agenda*. *National Academies Press* (The National Academies Press, 2019).
25. McQueen, N. *et al.* A review of direct air capture (DAC): scaling up commercial technologies and innovating for the future. *Progress in Energy* (2021).
26. Baker, S. E. *et al. Getting to neutral: options for negative carbon emissions in California*. (2019).

27. McQueen, N. *et al.* Cost Analysis of Direct Air Capture and Sequestration Coupled to Low-Carbon Thermal Energy in the United States. *Environmental Science & Technology* **54**, 7542–7551 (2020).

28. Kwon, H. T. *et al.* Aminopolymer-Impregnated Hierarchical Silica Structures: Unexpected Equivalent CO_2 Uptake under Simulated Air Capture and Flue Gas Capture Conditions. *Chemistry of Materials* **31**, 5229–5237 (2019).

29. What is direct air capture and storage? | Climeworks. https://climeworks.com/what-is-direct-air-capture-and-storage.

30. Beltran, A. M. *et al.* When the Background Matters: Using Scenarios from Integrated Assessment Models in Prospective Life Cycle Assessment. *Journal of Industrial Ecology* **24**, 64–79 (2020).

31. Thomson, R. C., Chick, J. P. & Harrison, G. P. An LCA of the Pelamis wave energy converter. *Int J Life Cycle Assess* **24**, 51–63 (2019).
