# Supplementary material for: Environmental trade-offs of direct air capture technologies in climate change mitigation toward 2100
Source: Nat Commun. 2022 Jun 25;13:3635. doi: 10.1038/s41467-022-31146-1 (PMC9233692; doi:10.1038/s41467-022-31146-1)
Supplement: Supplementary file 2 — Description of Additional Supplementary Information [file 41467_2022_31146_MOESM2_ESM.pdf]

Description of Additional Supplementary Data

Title: Supplementary Data 1 - Code and Data

Description: • Code folder: This folder includes the Python and R code of this study. The Python code is mainly used to import and analyze IMAGE raw data, develop and run the LCA model. R-code is used to post-process the IMAGE and LCA results and make figures. • Data folder: This folder includes the raw IMAGE raw data and the LCI data of some electricity generation technologies (wave, fossil fuel with CCS), biomethane heat supply, and amine-base sorbent.
